# Discrete-Particle Model to Optimize Operational Conditions of Proton-Exchange Membrane Fuel-Cell Gas Channels

**DOI:** 10.1021/acsaem.1c01391

**Published:** 2021-09-22

**Authors:** Daniel Niblett, Stuart Martin Holmes, Vahid Niasar

**Affiliations:** Department of Chemical Engineering and Analytical Science, University of Manchester, Manchester M13 9PL, U.K.

**Keywords:** fuel cells, two-phase flow, regime map, discrete particle model, gas channels, volume of fluid

## Abstract

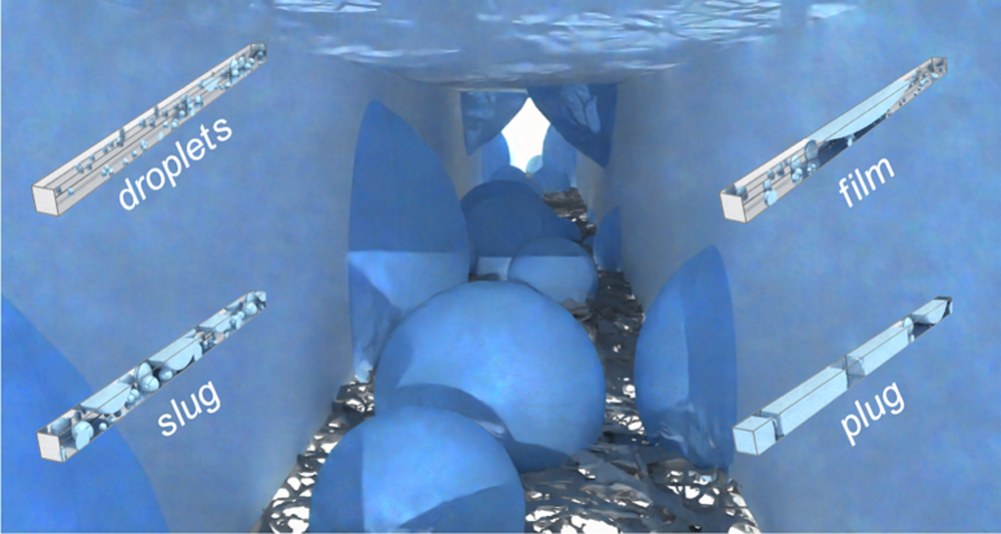

Operation of proton-exchange membrane fuel cells is highly deteriorated by mass transfer loss, which is a result of spatial and temporal interaction between airflow, water flow, channel geometry, and its wettability. Prediction of two-phase flow dynamics in gas channels is essential for the optimization of the design and operating of fuel cells. We propose a mechanistic discrete particle model (DPM) to delineate dynamic water distribution in fuel cell gas channels and optimize the operating conditions. Similar to the experimental observations, the model predicts seven types of flow regimes from isolated, side wall, corner, slug, film, and plug flow droplets for industrial temporal and spatial scales. Consequently, two-phase flow regime maps are proposed. The results suggest that an increase in water accumulation in the channel is related to the increase in the water cluster density emerging from the gas diffusion layer rather than the increased water flow rate through constant water pathways. From a modeling perspective, the DPM replicated well volume-of-fluid channel simulation results in terms of saturation, water coverage ratio, and interface locations with an estimated 5 orders of magnitude increase in calculation speed.

## Introduction

Polymer electrolyte fuel cells (PEFCs) can utilize hydrogen produced by renewable energy through water electrolysis.^1^ This is important for transportation where emissions damage health and the environment.^2,3^ Understanding the multiphase processes that occur in PEFC is important for the design of new materials which can improve performance and lifetime.

**Figure 1:**
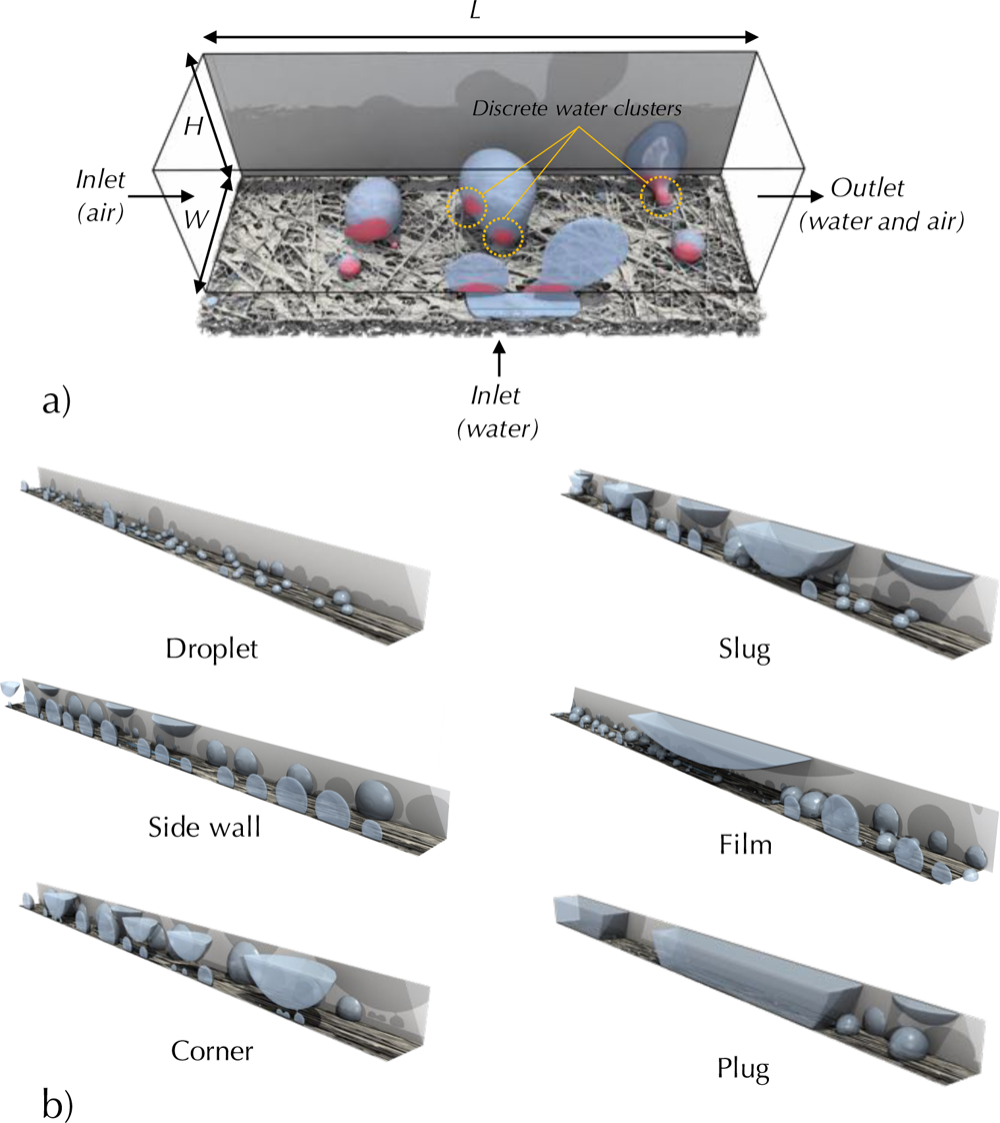
(a) Three-dimensional visualization of water appearing from the GDL and interacting with the channel walls. (b) visualization of discrete particle model (DPM) model results, showing water configuration for different two-phase flow regime descriptions of droplet, side wall, corner, slug, film, and plug flows.

During operation, spatial distribution of reactants (oxygen) across the cell is affected by the gas channel configuration and the presence of liquid water (products). Water emerges from the gas diffusion layer (GDL),^4,5^ creating a complex two-phase flow system, as shown in Figure 1a. PEFC channels (flow fields) can vary in configuration: serpentine, parallel, or interdigitated arrangements.^5^ Generally, these air supply channels are long (>0.1 m) and have square or rectangular cross sections (<1 mm^2^).^6,7^ This creates a system where processes occur at large spatial and temporal scales (e.g., a serpentine channel length of 0.655 m, where water emerges in the channel over 130 min^8^).

Two-phase flow in channels is encountered in other applications, including PEWE^9^ and boiling heat transfer in microheat exchangers used to cool electronic equipment.^10,11^ During PEFC operation, air and water have orders of magnitude difference in velocity. To characterize the effect of these variables on the flow pattern, two-phase flow regime maps for PEFC have been developed.^12−16^ However, quantitative data are difficult to obtain from the reflective materials of the GDL and water, including the two-dimensional view in transparent operating fuel cells.^4,5^

### Channel Flow Regimes

A variety of flow regimes can be observed in PEFC channels. Figure 1 demonstrates the notable flow types that are produced in the channels during operation, which form the basis of our model development.

The “droplet flow” regime occurs at high air velocity, resulting in droplet detachment with smaller diameters.^5,12,13,17^ Consequently, droplets are flushed away without significant accumulation or coalescence. The “side wall flow” is a subset of the droplet flow regime where droplets either emerge close to the side wall or grow large, eventually attaching to the channel side walls if the air velocity allows. In this regime, droplets spread on the two side walls and may be considered as annular flow.^16^ “Corner flow” occurs when fluids reach the top channel corner where capillary action pulls the fluid into the corner, detaching it from the GDL surface. In a 2D view, this may also be considered as annular flow. “Slug flow” occurs when water bridges across between the two side walls of the channel, which causes significant blockage of the channel. This occurs due to the accumulation of water and from the strength of capillary forces that maintain the slug structure at low air velocity, preventing the deformation into a film.^18−20^ “Film flow” is a subset of slug flow, where the fluid interface is deformed by the external forces of air because the capillary forces cannot maintain the surface curvature.^21^ “Plug flow” occurs when the water completely blocks the pathway for air to flow, as shown in Figure 1 and usually occurs at low air velocity where the momentum exchange is not large enough to deform the interface. The emergence of slug and plug flow (usually at low air velocity) can cause pressure fluctuations, often reaching magnitudes larger than the average pressure drop from operating at higher air velocity, as shown in experiments.^11,22^ Experiments have shown that increasing the water flow rate causes pressure fluctuations to occur more frequently,^23^ which may be related to the increased slug and plug flow frequency and also the periodic removal or water.

Regime maps and pressure drop correlations developed outside of the fuel cell devices may not be applicable for PEMFC channels because of the interaction with the GDL and different wettability walls compared to transparent channels.^4^ Water and airflow rates varied in a microchannel model of a PEMFC^12^ showed a transition from slug to droplet (5 m s^–1^) to film flow (30 m s^–1^) as the velocity increases. Other regime maps have shown the transition between slug, droplet, and film flow to exist between 1 and 3 m s^–1^.^14,15,21^

Alternatively, the wettability and length of the channel can also contribute to the change in flow regimes.^13,16^ Consequently, the prediction of air–water two-phase flow regimes is uncertain because the transition between the flow regimes is gradual as shown by the presence of the same droplet types in each regime in Figure 1. Moreover, the classification of the flow regimes is subjective, and an efficient computational method that can explicitly track the fluid distribution along the channel is desired.

### Modeling of Two-Phase Flow in Gas Channels

A review of two-phase flow models^4^ showed that the majority of models employed are the volume of fluid (VoF) and lattice Boltzmann method. Interface resolving simulation techniques such as VoF are valuable for developing insights and understanding into local-scale phenomena that can be used for upscaling and simplified models.^4^ However, they are limited by their computational cost and cannot be used to study PEFC operating scenarios.

For example, a coupled 1D membrane electrode assembly (MEA) and 3D VoF channel model (0.1 m, 20 injection pores)^6^ required 2 weeks per simulation run. Other studies that couple VoF and MEA equations^24^ used an accelerated water injection rate to account for the computational cost. Although, this still required 2 months on eight CPUs for a single case. VoF can replicate μCT data for water emergence from the GDL and detachment in the channel with an air velocity of 15 m s^–1^,^25,26^ with good replication of detachment size and shape. Furthermore, VoF has also shown its accuracy at modeling water flow inside the porous network.^27^

An accelerated water flow rate is used to reduce computation time, which is acceptable for capillary dominated flow in the GDL and for single droplets in the gas channel.^25,26^ However, it may not be valid for flow in the channel with multiple coalescence events. This has been illustrated by ref (6) which showed that at above ×100 the water generation rate, the distribution and saturation of water in the channel changed compared to ×10. The majority of studies for channel flow in PEFCs use analytical force balance calculations on single droplets to determine the detachment diameter at varied air velocity.^17,28−34^ These studies have been valuable for insights into developing the analytical force balances used in this study as they validate their predictions with experimental results^17,28,35^ with acceptable agreements. However, coalescence and attachment to the walls (where droplet geometry changes) have not been considered. These studies collectively show that increasing the air velocity decreases the detachment droplet height for a single droplet.

A phenomenological model^36^ accounted for droplet and corner film conditions in channels with momentum exchange with the air but did not track the droplets explicitly. In another study,^37^ the gas channel was coupled to a MEA where droplet dynamics were simulated including transport of oxygen, allowing accurate prediction of PEFC operation compared to experimental data.

Mechanistic approaches such as using Lagrangian methods can facilitate control of droplet collision phenomena.^4^ In such a framework, collisions involving only two water droplets have been studied extensively so that the outcome (coalescence or bouncing) can be predicted^38^ (in PEFCs, the majority of collisions result in coalescence). Lagrangian methods have been used for droplet interaction in sprays^39^ and also for dispersed bubbly flows.^40^ In our study, a similar approach is utilized, treating the water droplets as discrete particles with water source points representing the scenarios shown in Figure 1a.

This paper addresses the following questions using a mechanistic DPM:

- What conditions lead to the development of performance critical flows?
- How does the two-phase flow dynamics develop over large spatial (cm) and temporal scales (minutes)?
- What is the role of operating conditions (i.e., air velocity, current density, and wettability) in water distribution?
- What is the effect of GDL boundary conditions on water in the channel (e.g., density and distribution of water clusters)?

## Methodology

**Figure 2:**
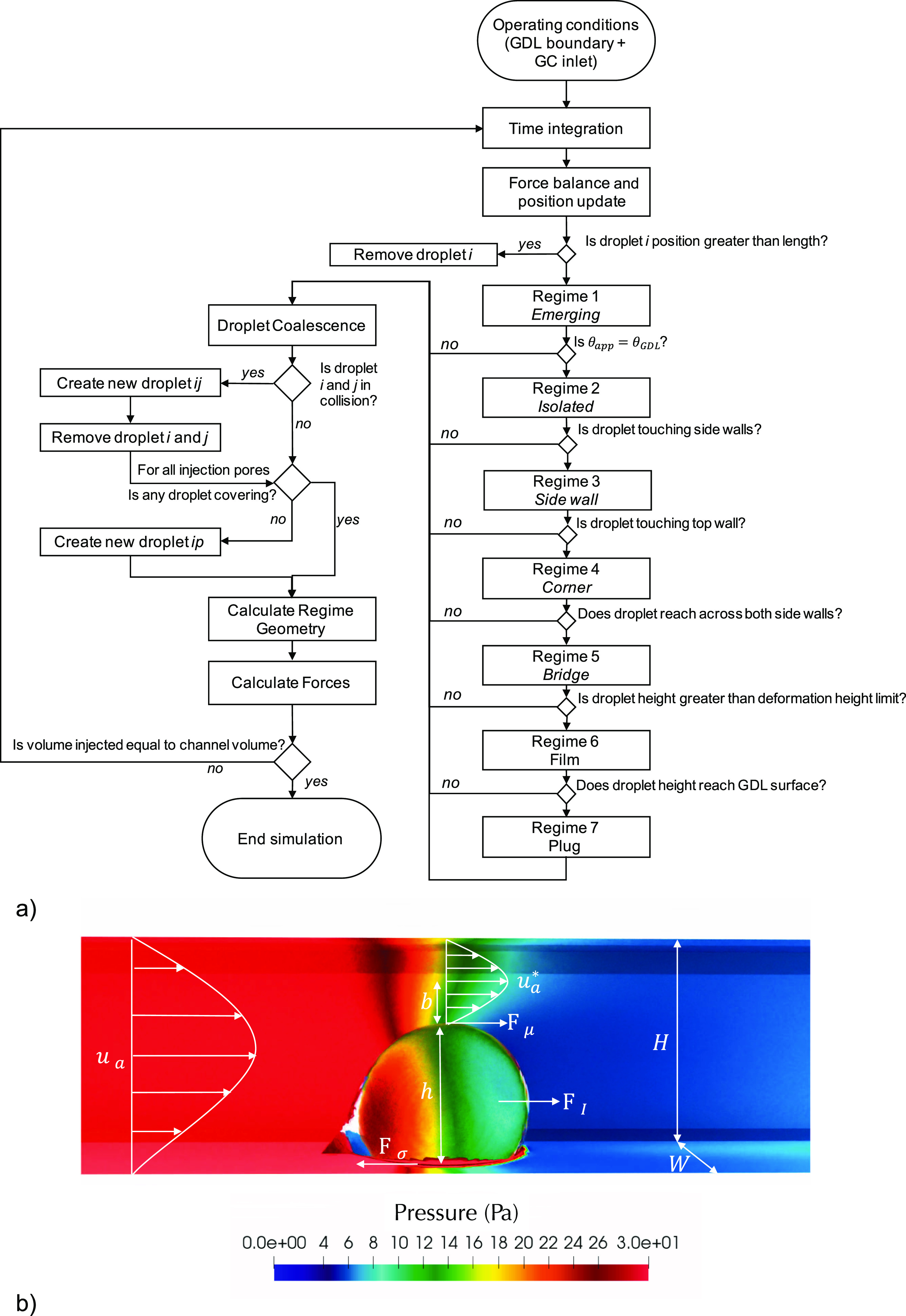
(a) Process flow chart for the DPM used to model water flow in PEMFC channels. (b) Schematic of the force balance on a droplet in a channel used in this study; pressure in the channel is increased by the presence of the droplet. The simulation highlighted was performed using VoF in OpenFOAM.^41^ All geometrical configurations for regime 1–7 are shown in Figure 3.

This DPM accounts for water flow in PEFC channels with different wettability walls and water injection from discrete sources on the GDL surface (Figure 1a). The model complexity was reduced into a process flow chart shown in Figure 2a.

The Lagrangian mass and momentum balances are solved using an explicit method. Each droplet regime is verified by different criteria shown in Figure 2a. During collision, a new droplet is created and the parent droplets are removed. If droplets disconnect from water cluster points on the GDL surface, a new droplet with zero volume is created. For each water regime, the geometry is updated depending on the water configuration (changing the force balance calculations). The simulation is terminated if the volume of water injected is equal to the total channel volume. This provided dimensionless comparison between channels with different water injection rates.

### Model Assumptions

The flow of air and water is incompressible flow at isothermal conditions with no phase change. Airflow is assumed to be fully developed laminar flow along the length of the channel. Deviation in shear stress caused by a square cross section is accounted for using modified Poiseuille equations.^42^ Water mass and momentum are tracked in a Lagrangian method with the center of mass and radius determined by each droplet regime classification. Three-way coupling is used in this study, and droplet interaction between the channel, other droplets, and the air phase is considered. The model was developed for wall contact angles above 45°, negating the formation of water filaments.^43^ Transport of species and consumption of oxygen along the channel is not considered. The mass conservation of air along the channel length (*z*) is
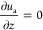
1

Air pressure loss in the channel is estimated from the viscous shear loss to the channel walls and momentum exchange with droplets. Air pressure drop along the channel as a function of channel geometry and air viscosity (μ_a_) was estimated as^42^
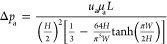
2where *H* is channel height and *W* is the channel width. If water droplets are present in the channel, pressure drop of the droplet due to the droplet Δ*p*_*i*_ is added.
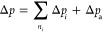
3

The pressure drop from frictional losses Δ*p*_a_ and pressure loss from momentum exchange with droplet Δ*p*_d_ is calculated using eqs 2 and 12, respectively, to be introduced later.

### Water Conservation Equations

Discrete water clusters in the GDL are assumed to be fully formed following the observation in μCT experiments.^44^ The water flow rate *Q*_w_ is distributed across all injection locations defined in the channel to determine the water injection velocity at each pore.
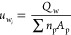
4

The change of volume of a droplet *V*_*i*_ is determined by the number of injection sites *n*_p,*i*_ it is connected to
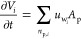
5

A visualization of multiple injection sites contributing to one droplet can be seen in Figure 1a. During droplet coalescence between droplet *i* and *j*, the water volume is conserved by addition.

### Force Balance on a Single Droplet

Airflow around a confined droplet causes the forces shown in Figure 2b. At the scale of the fuel cell channel, surface adhesion, inertial, and viscous shear forces are of similar magnitude.^10^ Droplets are pinned in place until the drag force *F*_D_ overcomes the adhesion force *F*_σ_. The magnitude of the adhesion force is increased if the droplets are connected to a water cluster inside the GDL.^33,45^ By approximating each droplet as a discrete particle, the momentum balance solved explicitly for each droplet *i* is
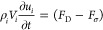
6

The predicted droplet velocity *u*_*i*_ obtained from eq 6 is used to determine the droplet position in the same time step. It is assumed that the droplet is connected to water in the GDL until the droplet moves a distance *R*_i_ + *R*_p_ away from water injection sources. After this point, the droplet is disconnected from the GDL water cluster. The drag force *F*_D_ consists of the inertial *F*_I_ and fluid–fluid viscous shear *F*_μ_ forces acting on the droplet as well as the opposing fluid–solid viscous shear *F*_μ,s_ forces acting on the fluid–solid surfaces.
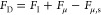
7

Air accelerates around droplets, converting pressure energy into kinetic energy, resulting in a pressure drop acting on the droplet surface normal to the direction of airflow. This pressure loss in the air can be visualized by the sharp color gradient in Figure 2b, resolved using VoF in OpenFOAM.^41^ Additional information regarding the derivation of the forces in eq 7 is presented in Supporting Information.

The inertial force was predicted using the mass conservation of air (eq 1) to predict the velocity of air in the cross section of the droplet, combined with the droplet cross-sectional area normal to the airflow *A*_c_. The accelerated air velocity was determined as *u*_a_^*^ = *fu*_a_, where . Using the Bernoulli equation, the inertial force acting on the droplet cross section was estimated as
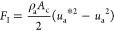
8

The viscous shear force *F*_μ_ is caused by the viscous shear stress acting on the fluid–fluid interfacial area *A*_s_ parallel to the direction of flow. This was estimated by the shear stress acting on walls during planar Poiseuille flow between the maximum droplet dimension into the channel domain (i.e., droplet height *h*)
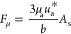
9where *b* is the approximate thickness of the viscous boundary layer in laminar flow shown in Figure 2b, which for the isolated droplet regime is *b* = *H* – *h*/2. The fluid–solid viscous shear force *F*_μ,s_ appears during water movement and is calculated assuming a linear gradient in water velocity from the center of the droplet to the channel walls (no slip condition)
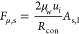
10where *A*_s,l_ is the surface area between the solid and liquid and *R*_con_ is the contact radius of the droplet on the channel or GDL walls.

The adhesion force *F*_σ_ appears due to the resistance of the external drag force. This restricts the drag force, contributing to droplet movement until it can overcome the adhesion force. The adhesion force acts on the liquid–solid contact line *L*_σ_ normal to the direction of flow.^28,31,32,46^ It is estimated based on advancing θ_A_ and receding θ_R_ contact angles formed by the deformation of the interface
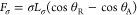
11where σ is the interfacial tension. Deformation of droplets under external forces was not considered in this study. A fixed contact angle hysteresis of 25° was used in this study to estimate the effect of a receding contact angle for droplets that are pinned to injection pores and 1° for droplets that are not attached to pores.

The contribution of each droplet to the overall air pressure drop in the channel shown in eq 3 is calculated as the summation of eqs 8 and 9
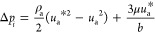
12

### Droplet Tracking and Coalescence

Three different tracking algorithms are used within the DPM:

1. Droplet *i* to droplet *j* collision (coalescence)
2. Droplet *i* to wall collision (wall attachment)
3. Droplet *i* to injection source p overlap (water volume growth)

These events are assumed instantaneous within the same time step. Accounting for the real dynamics would require a computationally demanding method such as VoF which is inefficient at large timescales.^4^

### Coalescence Algorithm

When two droplets come into close contact, the thin film of air between them is removed and water interfaces merge together. This is simplified by instantaneous birth and death of droplets, similar to the method used for DPM spray modeling.^39^ The momentum exchange during collisions of droplets with different velocity and mass is conserved by an inelastic collision.

### Injection Algorithm

The mass conservation for each droplet used in eq 5 requires the number of injection points each droplet covers *n*_p_ to be identified. By comparing location of injection points, the center of the droplet and its radius, the total number of injection points covered by droplet *i* is calculated. After disconnection of a droplet from the injection point (by either wall attachment, coalescence, or by the action of air), new droplets will start to grow in its place.

### Water Regimes in the Channel

**Figure 3:**
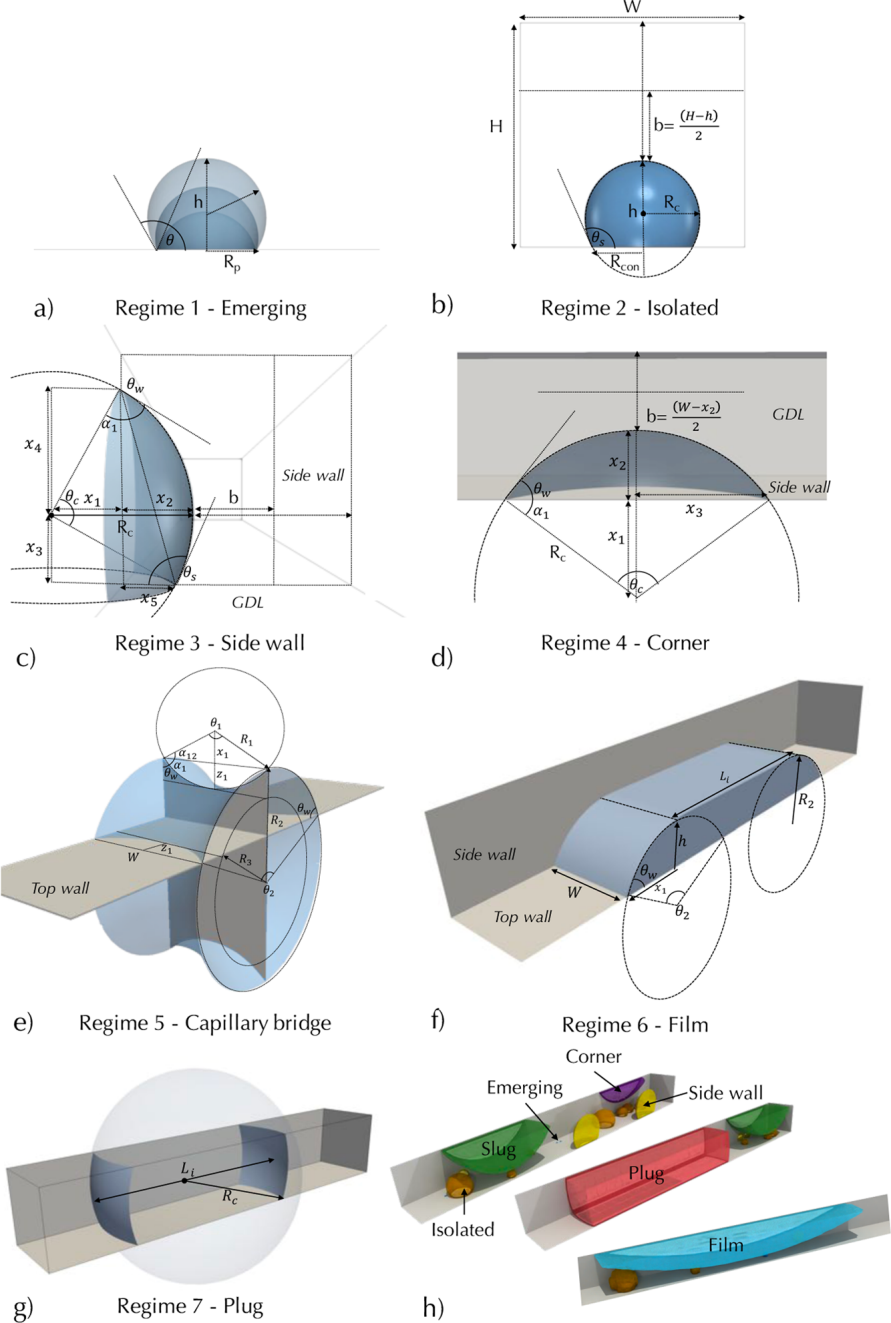
Schematics used for derivation of geometric regime properties for (a) emerging, (b) isolated, (c) side wall, (d) corner, (e) truncated capillary bridge (slug flow), (f) film flow, and (g) plug flow. (h) Shows a volume-mapped DPM output for each of the regimes presented above [capillary bridge is termed slug flow, and the concave shape is a visual representation of *R*_3_ in (e)].

Seven geometrical configurations (regimes) are assumed for water droplets, depending on the GDL and channel wall surface wetting conditions. These are termed as emerging, isolated, side wall, corner, truncated capillary bridge (slug), film, and plug flows. Evolution of these regimes within the channel is tracked as shown in Figure 3h. The truncated capillary bridge in renderings is shown with a convex surface due to the visualization limitations (clipping radius of curvature by the channel walls).

The simplification of interface geometry is inspired from the visualization of the interfaces produced by the VoF method^6,26,47^ and from transparent fuel cell channel experiments^8^ where capillary forces determine the droplet shape.^9,48^ Based on the volume of each droplet *V*_*i*_ and the contact angle of the channel walls, the radius of curvature and geometric center was determined using the schematics in Figure 3a–g. The top wall regime, where droplets can attach directly to the top wall of wide rectangular channel dimensions was not considered in this study because it is unlikely to occur in square channels. Additional information regarding the derivation of each regime can be found in Supporting Information.

### Regime 1—Emerging Droplet

Droplets emerge from the GDL following a constant contact radius (CCR) growth. The CCR is defined as the radius of the water injection pore shown in Figure 3a. The apparent contact angle the spherical cap makes with the surface is tracked until it is equal to the GDL contact angle θ_s_. This occurs as an inflating sphere and can be visualized in Figure 3a. Assuming a spherical cap shape with no deformation, the volume of the droplet *V*_*i*_ can be expressed as
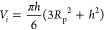
13which is solved for the height of the droplet *h* using an iterative method, where *R*_p_ is the radius of the pore. The radius of curvature is calculated from a different formula for the volume of a spherical cap using the droplet height.

The collision radius used in this regime is the pore radius *R*_p_. This was used to remove the effect of large curvature values (i.e., flat water surface) with low apparent contact angles which caused nonphysical coalescence. The apparent contact angle θ_app_ the interface creates with the surface is
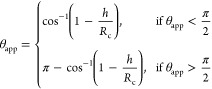
14When θ_app_ > π/2, the radius of curvature is used for the droplet collision detection. All droplets in regime 1 with θ_app_ = θ_s_ will transition to regime 2 (isolated), as shown by Figure 2. This is the transition between the CCR and constant contact angle (CCA) modes of growth.

The parameters required for the force balance, radius of curvature, cross-sectional area, interfacial area, and contact length are
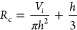
15
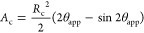
16
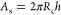
17
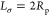
18

The assumptions of CCR and CCA modes were used to simplify the model for a flat porous surface. In reality, a droplet is pinned to individual fibers, so the triple contact line will move around each fiber to maintain local contact angle during droplet growth. This will continue until the curved interface attaches to neighboring fibers. This essentially transitions the droplet into the CCA mode.

### Regime 2—Isolated Droplet

Isolated droplets are a continuation of volume expansion from regime 1. Droplets grow with a CCA with dimensions shown in Figure 3b. The droplet volume and contact angle are used to determine the radius of curvature for the spherical cap
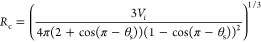
19

The radius of curvature of this regime is used to calculate the droplet properties required for the balance of forces acting on the droplet using the same set of equations as regime 1, replacing *R*_p_ with *R*_con_. The cross-sectional area normal to the airflow is
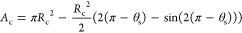
20

### Regime 3—Side Wall Droplet

When a droplet collides with the side walls, it will be pulled to the water-wet wall by the capillary action. This droplet shape is shown in Figure 3c. The radius of curvature of this shape was derived as
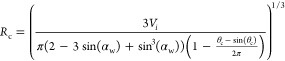
21where θ_c_ = 2(π – θ_s_) and α_w_ = (π/2 – θ_w_). When the droplet height (*h* = *x*_3_ + *x*_4_ shown in Figure 3c) in the *y*-direction is greater than or equal to the height of the channel *H*, the droplet transitions into regime 4, corner droplet. The parameters required for the force balance are
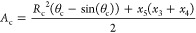
22
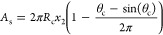
23
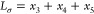
24where the dimensions *x*_2_, *x*_3_, *x*_4_, and *x*_5_ are shown in Figure 3c and the equations are defined in Supporting Information.

### Regime 4—Corner Droplet

The interface geometry of this shape is shown in Figure 3d. The method for predicting the radius of curvature truncates a spherical droplet based on the wall contact angles. Therefore, the radius of curvature was predicted as function of the droplet volume and wall contact angle
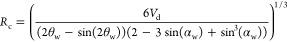
25where α_w_ = (π/2 – θ_w_). If the dimensions of the droplet across the channel (*x*_2_ shown in Figure 3d) are greater than the channel width, the droplet will move into regime 5, truncated capillary bridge. The properties required by the force balances are
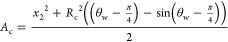
26
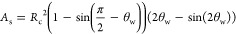
27
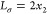
28

### Regime 5—Truncated Capillary Bridge

A truncated capillary bridge is formed between the channel side and top walls when water in the corner (regime 4) reaches across the channel. This regime is usually termed slug flow and is shown in Figure 3e. It has two radii of curvature, related to the channel width *W*, channel wall contact angle θ_w_, and volume of the water cluster *V*_*i*_, as shown in Figure 3e. The radius of curvature of the interface in the middle of the channel *R*_1_ is
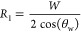
29which is used to calculated dimension *z*_1_ in Figure 3e.
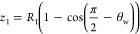
30

The secondary radius of curvature *R*_2_, shown at the channel walls is
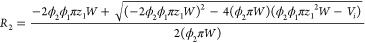
31where the area ratio parameters ϕ_1_ and ϕ_2_ are
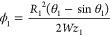
32
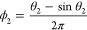
33with θ_1_ = π – 2θ_w_ and θ_2_ = 2θ_w_. When the height of the droplet (*h* = *R*_2_ – *z*_1_) is equal to the channel height *H*, the droplet is considered to block the flow path and begins to move at the velocity of the air (regime 7—plug flow). Parameters required by the force balance in this regime are approximated as
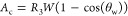
34
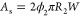
35
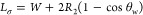
36

### Regime 6—Film Flow

If the droplet is attached to the top wall, the capillary bridge can deform depending on the airflow conditions around the droplet. This liquid slug has a height that is limited by the compression of the air (found by a force balance, discussed later) and instead elongates along the length of the channel, as shown in Figure 3f and in blue in Figure 3h. This uses the same shape as the truncated capillary bridge (regime 5), although at the cross section, the interface becomes parallel to the top channel wall. The primary radius *R*_1_ is calculated using eq 29, and the secondary radius *R*_2_ shown in Figure 3f is
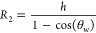
37

After derivation, the length of the film along the channel *L*_*i*_ is estimated as
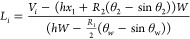
38where θ_2_ = (2θ_w_ – π/2). The length of the film along the channel is used to estimate an equivalent radius of curvature (based on the equation for a circular segment) to use for visualization and droplet coalescence shown by Figure 3h. The parameters required by the force balance in this regime are approximated as *A*_c_ = *hW* and *A*_s_ = *L*_*i*_*W*, where the adhesion length is also represented by eq 36.

### Regime 7—Plug Flow

Plug flow only forms if the external flow conditions prevent film formation and if the droplet slug grows large enough in volume. To ensure mass conservation in the air phase, the water plug must move at the volumetric flow rate of the air *Q*_a_ resulting from the one-dimensional flow in the channel. A simple approximation for the length of the plug as shown in Figure 3g is estimated as *L*_*i*_ = *V*_*i*_/*HW*, where the radius of curvature is estimated as *R*_c_ = *L*_*i*_/2 and the geometric center is in the middle of the channel. This approximation is valid because the formation of a plug will flush the system of all droplets downstream. Variation in the plug shape will not have a significant effect on coalescence events.

This model does not consider flow through the porous GDL. Coupling the DPM to a flow solver for the channel and GDL will allow correct implementation of this regime. If the resistance to move the plug is greater than the resistance for flow through the GDL or an adjacent channel, then it will remain in place. This could be handled by a hydrodynamic network model approach.^49^

### Capillary Bridge Deformation

To estimate the height at which interface deformation becomes significant (required to solve eq 38 for regime 6—film flow), a balance of forces for Laplace pressure, which determines the interface shape, and external pressure (surface viscous shear stress and inertial) is
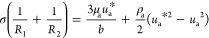
39

This was developed for a cylindrical droplet slug in the channel (between two side walls and the top wall) and assuming only one radius of curvature (along the channel), using a cylindrical cap assumption,^20^ as depicted in Figure 3f. Using the calculated air velocity, capillary number (*Ca*) (ratio of viscous to capillary forces), and Weber number (*We*) (ratio of inertial to capillary forces) are defined as
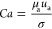
40
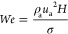
41

The balance of forces is solved using an iterative method to find the droplet height *h* at which the pressures are equal
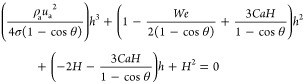
42

**Figure 4:**
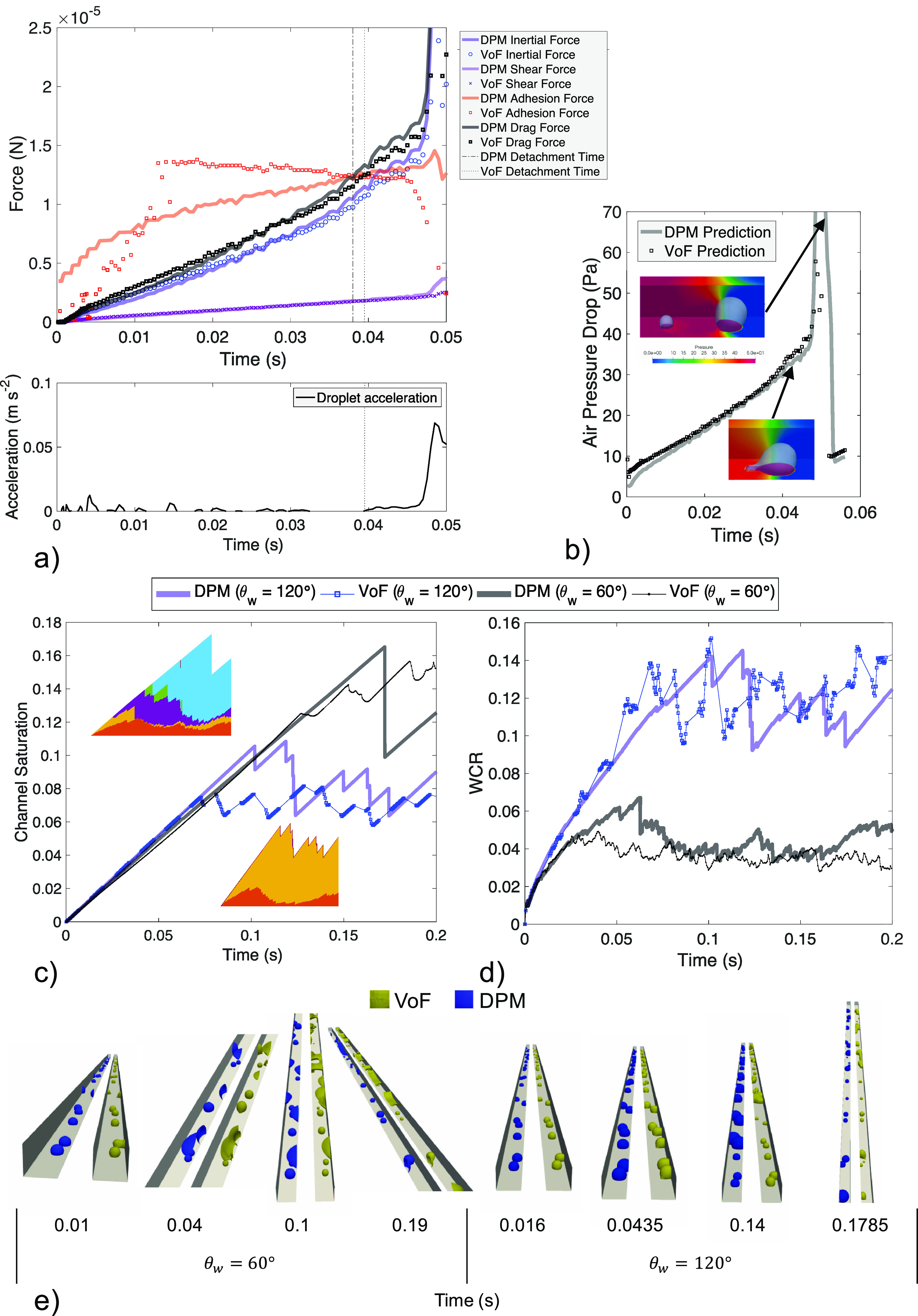
(a) Extracted (CFD) and predicted (DPM) forces and acceleration for a single droplet detachment. (b) Predicted pressure drop. (c) Comparison between DPM (shaded lines) and VoF simulations (points) for channel saturation at varied wall contact angles (transient regime profiles shown). (d) Prediction of the WCR. (e) Comparison of 3D renderings of fluid configurations at different times for two wall contact angles of 60 and 120° (VoF = yellow and DPM = blue).

This geometry is shown in Figures 1, 3h, and 4c with film development. This can be improved by accounting for gravitational body force and the radius of curvature between the two channel walls.

### Boundary Conditions and Time Stepping

Droplets emerge into the channel at locations and rates determined by the distribution of water clusters within the GDL.^22,26,28,33,50^ Micro X-ray-computed tomography (μCT) experiments show the formation of discrete water clusters in the GDL^44,51,52^ and spherical droplets on the GDL surface in the channel.^53^ Therefore, the water injection rate is into each droplet is specified by the water source velocity *u*_w_*i*__ or by the operating current based on the available active area (*WL*)
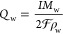
43where *I* is the current and *M*_w_ is the molecular mass of water. The water injection velocity per pore is the same along the length of the channel if all the pore radii are the same. The heterogeneity of water flow rates along the channel was not considered as the transport equation for oxygen flow was not solved. The inlet velocity of air was determined using the air volumetric flow rate *Q*_a_
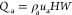
44where ρ_a_ is the density of air and *H* and *W* are the height and width of the channel, respectively. The air velocity is either set or determined by the reaction stoichiometry ν, operating current *I*, and the mole fraction of oxygen in air *x*_O_2__. This was determined by converting the oxygen molar consumption rate along the active area (*WL*) to the volumetric flow rate using the ideal gas law
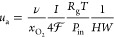
45where *P*_in_ is the inlet pressure (1.2 bar), *T* is the inlet temperature (60 °C), and *x*_O_2__ is the oxygen mole fraction in air (0.21).  is the Faraday constant (96,485 A mol^–1^), and *R*_g_ is the universal gas constant (8.314 J mol^–1^ K^–1^).

An adaptive time stepping scheme was used to maximize computational efficiency and to ensure that no collision event is missed. Two criteria were used to determine the maximum time step Δ*t* (s): (a) the maximum velocity of droplets, air, and water source (*u* = max[*u*_*i*_, *u*_a_, *u*_w_*i*__]): Δ*t* = 0.1*H*/*u* and (b) closeness of the drag force to the adhesion force (indicating the point of instability)
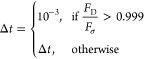
46

### Operating and Material Parameters

The majority of channel-operating and material parameters can be varied to study their effect on the distribution of water in a straight channel, as shown by Table 1. From VoF simulations and literature data, the cluster density was estimated to be between 5 and 10 water clusters per mm^2^.

**Table 1:** List of the Boundary Conditions Used to Generate the Results from the DPM

parameters	typical values
channel dimensions (*H* × *W* × *L*)	0.001 × 0.001 × 0.1 [m]
air injection velocity	0–32 [m s^–1^]
water injection velocity	10^–5^–1 [m s^–1^]
injection source density	1–36 [n mm^–2^]
injection source location	
channel wall wettability (60°)	45–180 [deg]
GDL surface wettability (120°)	45–180 [deg]
water properties (μ, ρ, σ)	10^–3^ [Pa s], 1000 [kg m^–3^], 0.072 [N m^–1^]
air properties (μ, ρ)	1.9 × 10^–5^ [Pa s], 1 [kg m^–3^]

### Channel Performance Metrics

The configuration of water in the channel is an important factor.^6,7,54^ Water occupying the GDL surface can lead to increased resistance for oxygen to diffuse into the GDL and subsequently to the catalyst layer. Using the geometric approximations for droplets, the water coverage ratio (WCR) is defined as
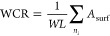
47where *A*_surf_ is the contact area between droplets and the GDL surface. Droplets can exhibit different distributions of droplet regimes over time, and therefore, a regime parameter fraction was created. This defines the fraction of water that is within a regime classification ϵ and is calculated as the volume of water droplets *i* in regime *r* divided by the total volume of water in the channel
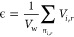
48

Visualization of the model outputs was developed as VTK using droplet radii and center of volume, which was processed in Paraview by clipping spheres by the channel walls. In these visualizations, the truncated capillary bridge shape (slug flow) is shown to be spherical (convex using *R*_3_ in Figure 3e), which is used for visualization purposes only. In the model, the real concave dimensions shown in Figure 3e are used. This is due to the limitations of the current visualization technique of clipping spheres. The DPM simulations and model was computed in MATLAB on 1 CPU (2.3 GHz Intel Core i5, 8 GB RAM). The VoF simulations were computed using 16 CPUs (Intel Xeon E5-2640 0@2.50 GHz).

## Results and Discussion

### Model Validation

In the absence of experimental validation (resulting from the difficulty in replicating unknown boundary conditions related to water clusters emerging from the GDL), the model was validated in three scenarios against VoF numerical simulations:

1. Single-droplet detachment (Figure 4a)
2. Static capillary bridges (Figure S3 in Supporting Information)
3. Long channel with 30 injection points (Figure 4c–e)

This approach is valid because VoF has been compared against X-ray CT data for droplet detachment and movement in fuel-cell gas channels^25^ and water percolation in GDLs.^26,27^ Since the droplet detachment times predicted by VoF are similar to experiments, the magnitudes of forces predicted by VoF are likely to be accurate. However, VoF has not been validated against large-scale flow field channels because of the unknown injection boundary conditions and difficulties in data extraction from transparent fuel-cell studies.^5^

### Transient Droplet Emergence and Detachment

The single droplet injection and detachment simulation shown in Figure 4a,b was performed in a square channel with the operating conditions *W* = *H* = 0.001 m, *L* = 0.005 m, *n*_p_ = 1, *u*_w_ = 0.1 m s^–1^, *u*_a_ = 5 m s^–1^, θ_s_ = 120°, and θ_w_ = 60°. These conditions are equivalent to a current density of 2694 A cm^–2^ and stoichiometry of 0.13. The accelerated conditions were used to decrease the computational cost of VoF, and thus, this scenario does not represent any situation in PEFCs but was used to validate the forces predicted by the DPM. This used a parabolic velocity injection condition of air based on the fully developed laminar flow of air at 5 m s^–1^. The water injection rate was 0.1 m s^–1^ through a 200 μm square inlet located in the center of the channel. The simulation contained 625,000 cells with a resolution of Δ*x* = 20 μm. The simulation was terminated after the first droplet detachment, requiring approximately 1 day of simulation time (16 CPUs—Intel Xeon E5-2640 0@2.50 GHz). Forces and air-phase pressure drop extracted from VoF and predictions made by DPM are presented in Figure 4a,b.

The comparison between the VoF and DPM forces show that for the transient growth of a droplet in the channel, the equations used within this study can predict the inertial and shear forces to an acceptable level. However, for the inertial force, it was necessary include a scaling factor of 1.22 to best fit the data, which is likely a result of the inertial acceleration of air around the droplet which does not homogeneously distribute over the channel cross section. Regardless, the point of instability is shown by the increase in droplet acceleration in Figure 4a, and the air pressure drop was able to be predicted by the DPM. The DPM has an acceptable level of accuracy relative to VoF simulations but at a fraction of the cost [0.01 s (DPM) to 1 day (VoF) simulation time].

### Comparison between the DPM and VoF for a Channel

A VoF simulation for a straight channel was developed (1 M cells, Δ*x* = 25–50 μm). Both VoF and DPM had identical initialization, with the following channel properties: *W* = *H* = 0.001 m, *L* = 0.1 m, *n*_p_ = 30, *u*_w_ = 0.1 m s^–1^, *u*_a_ = 3 m s^–1^, θ_s_ = 120°, and θ_w_ = 60° or θ_w_ = 120°. This corresponds to an accelerated equivalent current density of 4041 A cm^–2^. The comparison between the simulation methods is shown in Figure 4c for saturation, (d) for the WCR, and (e) for 3D interface visualization.

Figure 4c shows the predictive capability of the DPM to predict the transient saturation of the channel with different contact angles at a water injection rate of 0.1 m s^–1^ (i.e., accelerated by ×1000 compared to operating fuel cell conditions). In the DPM, there is a large reduction in saturation caused by the instantaneous removal of droplets, whereas in the VoF simulation, droplets gradually leave the channel with saturation calculated based on the volume fraction in the channel. Nevertheless, it is promising that the model can replicate a similar magnitude of fluctuating steady-state values. Figure 4d shows the WCR from the comparison between the DPM and VoF model, which shows a good level of replication at both wall contact angles.

The match between the WCR shown in Figure 4d provided further confidence in the DPM to predict geometric properties of droplet regimes as a function of wall contact angles. Visual comparison between the DPM and VoF is shown in Figure 4e. The similarities between the droplet reconstruction by both methods at different times and wall contact angles demonstrate a good level of accuracy in terms of droplet interface locations and positions, as well as general two-phase flow patterns in the DPM versus VoF model.

The simulation time of 0.2 s using VoF required approximately 3 weeks (16 Intel Xeon E5-2650 v2@2.60 GHz), where the mechanistic model required 8.3 s with a constant Δ*t* = 1 × 10^–4^ s. The speed of the model (×200,000 faster) is the key advantage of the DPM that outweighs the slight discrepancy in predicting two-phase flow properties in the channel. The DPM allows phenomenological analysis of two-phase flow dynamics in the channel at much larger spatial and temporal scales, which would be computationally infeasible using CFD methods.^4^

### Two-Phase Flow Regimes

The DPM can produce two-phase flow regimes similar to experiments as shown in Figure 1 for droplet regimes of isolated,^15,25,50,55^ side wall,^8,15,55^ corner,^51^ slug,^8,15^ film,^15,16^ and plug^20^ flows.

**Figure 5:**
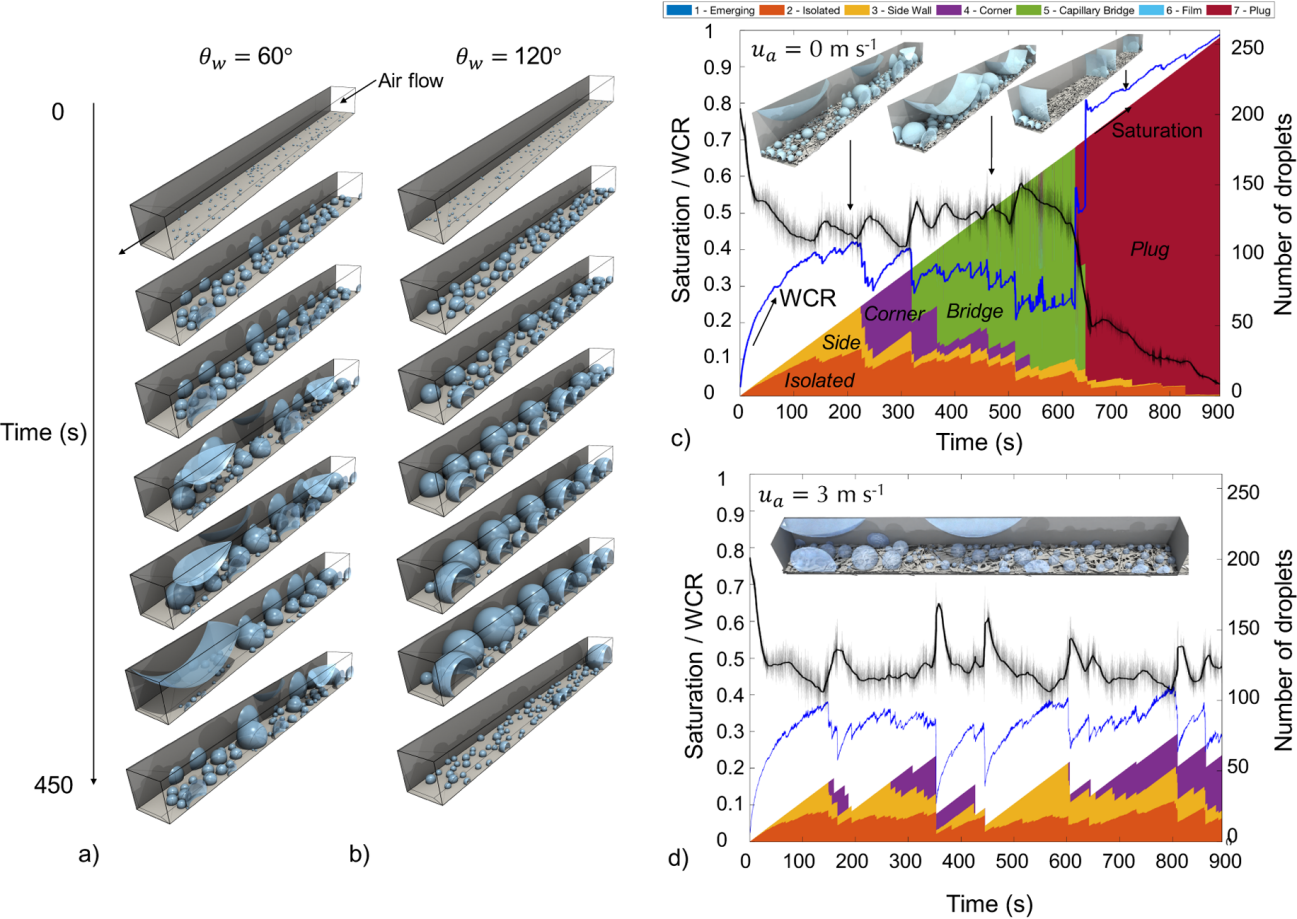
(a) 3D visualization of droplets emerging into a L = 1 cm channel at a current density of 1.2 A cm^–2^ from 0 to 450 s with θ_s_ = 120° and θ_w_ = 60°. (b) hydrophobic channel with θ_w_ = 120°, (c) saturation regime and WCR profiles (*u*_w_*i*__ = 2.19 × 10^–5^ m s^–1^ at 250 injection locations) with number of droplets tracked (mean = black line and variance = shaded lines) at no airflow conditions (*u*_a_ = 0 m s^–1^) and (d) with air velocity present (*u*_a_ = 3 m s^–1^).

Figure 5a,b shows the transient evolution of water (current density of 1.2 A cm^–2^) for wall contact angles of 60 and 120°, respectively, with water emerging from 250 discrete injection points. In Figure 5a, it can be observed that droplets spread on the side wall until enough volume has been accumulated to reach the corners. Corner droplets are disconnected from the water injection pores and do not change in volume until an adjacent droplet on the side wall grows large enough for coalescence. Once the volume is large enough to spread to the opposite channel walls, it forms a capillary bridge as seen in the later stages of time, which subsequently moves out of the channel due to the magnitude of drag forces. Alternatively with hydrophobic channel walls (Figure 5b), droplet growth does not reach the top channel corners before detachment.

The variance shown by the shaded lines in Figure 5c shows the time step-specific number of droplets, whereas the solid black line shows the smoothed time-averaged result. This was shown to improve visualization.

Figure 5c shows the regime fraction profile for the same channel in the absence of airflow. The droplets transition from isolated, side wall, corner, and to bridge droplets until plug flow formation occurs. Two separate plugs then continue to grow, as shown by the reduction of droplets (black line) from 150 to 50 during the transition between bridge and plug flow regimes. Simultaneously, the WCR (blue line) decreased with time due to the increased appearance of the capillary bridge (slug) regime. The sharp increase in the WCR occurs during the transition of capillary bridge droplets to plugs, which may cause oxygen reactant starvation during PEFC operation.

Alternatively, Figure 5d introduces air velocity (*u*_a_ = 3 m s^–1^), showing that as a consequence of momentum transfer, droplets are removed during the corner regime. Coalescence events did not form a bridge or plug across the channel due to removal of water. In both cases, the number of droplets present initially decreased from 250 to 120 droplets, which showed that droplets are connected to more than one injection source on average.

There is a periodic detachment and growth cycle exhibited when air is introduced into the system which is a phenomenon that was found in experimental channel studies.^12,20^ Mass conservation is shown to be enforced by the linear gradient in saturation, reaching one channel volume injection, as specified by the end point of the simulation process shown in Figure 2.

### Comparison with Experimental Data

Ideally, the DPM should be compared against experimental data. However, this would require knowledge of exact experimental conditions, such as droplet emergence density and high-resolution data.

The WCR is difficult to compare with experiments unless X-ray CT is employed. Droplets or films covering the top channel surface will lead to a higher apparent water coverage of the GDL surface.^15^ This effect is highlighted by the sharp jump in the WCR as bridge (slug) flow is transformed into plug flow, as shown in Figure 5c.

The single droplet detachment diameters predicted by the VoF and DPM in Figure 4a are of similar magnitude (400 μm) to that predicted by predictions and experiments of ref (21).

X-ray CT data of droplets in a channel show similar droplet regime characteristics as those shown in the visualization of Figure 5a.^55^

### Impact of Air and Water Velocity on Two-Phase Flow Regimes

**Figure 6:**
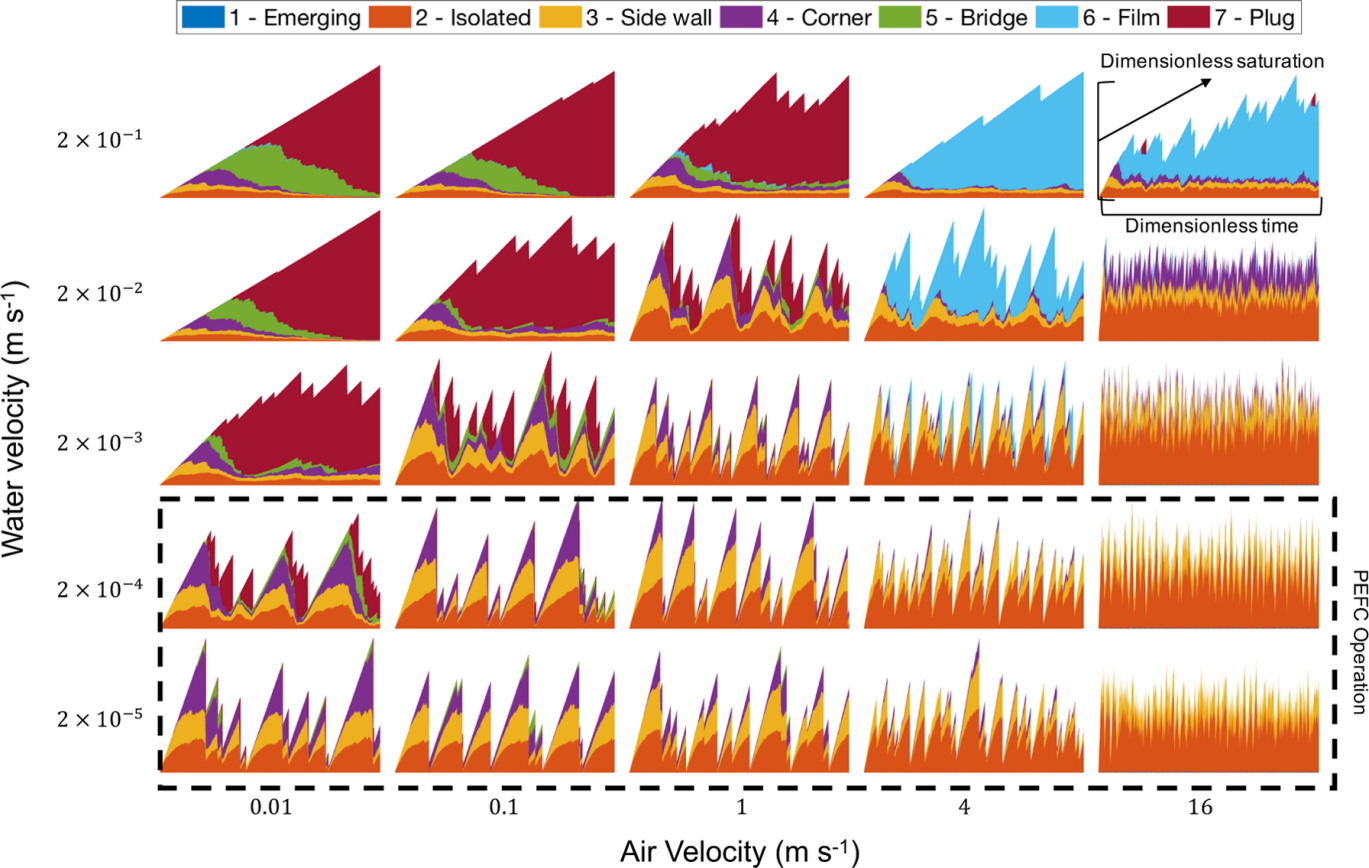
Saturation and regime profiles for 25 simulation cases, varying air velocity and water velocity with values corresponding to the marker (*x*, *y*) location. Channel dimensions are 1 mm × 1 mm × 0.2 m for the simulation period of one channel volume injection with water injection from *n*_p_ = 400. Fuel-cell operating space marked on the regime map by the dashed box corresponds to between 0 and 1.2 A cm^–2^. Each marker is scaled for dimensionless saturation and time (i.e., *t*/*t*_max_). The full 225 simulation cases are presented in Supporting Information, Figure S1.

To understand the effect of droplet coalescence and longer channel lengths, a sensitivity analysis study of injection rates was performed. In total, 225 simulations were performed by varying air velocity (*u*_a_ = 0.01–32 m s^–1^) and liquid injection velocity (*u*_w_ = 2 × 10^–5^ to 1 m s^–1^) with a different arrangement of injection locations for each run (*n*_p_ = 400). This range of flow rates covered the possible conditions found within operating PEFCs and also extended far above the operating range (i.e., in a fuel cell perspective current density from 0.1 to 10,000 A cm^–1^ and stoichiometry from 10^–3^ to 80) to test the flexibility of the developed DPM. The transient saturation regime profile for each simulation can be found in Supporting Information (Figure S1), and 25 are highlighted in Figure 6 for clearer visualization.

The markers in Figure 6 show the saturation regime profiles for each simulation case. The saturation regime map shows that there is a gradual transition between different operating modes for different water and air injection rates. At low air and water velocity, droplets transition through the regimes without resistance to growth until a capillary bridge and plug regimes eventually form. If the air velocity is large enough, these plugs refresh the system of all droplets downstream but stay in the system for only a very short amount of time compared to the total simulation time, especially at low water flow rates. At high water and low air velocities, each water cluster can reach the full range of regimes before detachment. Increasing the air velocity slightly allows the probability of slug coalescence to increase due to movement of clusters downstream. This caused a larger distribution of the plug flow regime in the intermediate region rather than at the low air velocity at which slug flow is more prevalent.

At an air velocity above approximately 2 m s^–1^, plug flow development is less prevalent; capillary bridges that would lead to plug flow now deform to film flow (shown by the blue areas in Figure 6) under the higher external stress from the airflow. This regime dominates at high air and water velocity because the films are regenerated by high water injection rates into the channel, causing further coalescence events to occur along the channel. In all cases, increasing the air velocity increases the frequency of detachment events (saturation peaks) and decreases the size of droplets for coalescence events. When the water flow rate is high and channel lengths are long, droplets have a greater chance of coalescence to larger slugs and plugs as they move down the channel.

Many simulation studies accelerate water injection rates to decrease the computational time and to avoid two-phase flow numerical errors.^5^ For a single droplet emergence location, this might be acceptable because there is no interaction between multiple droplets.^6,25,26^Figure 6 suggests that an accelerated water flow rate cannot be used to replicate the two-phase flow regime conditions at lower water flow rates demonstrated by the prevalence of plug and film flow regimes.

The length of the channel used in this study (L = 0.2 m) is long enough to provide sufficient statistics because it allows the development of plug flow, as well as the effect of plug flow in causing channel saturation flushing events. This is where all droplets downstream of the plug are quickly removed due to coalescence. The results in Figure 6 are plotted against dimensionless time for one channel volume of water injected. Multiple slow periodic processes are captured by the analysis which is highlighted by the bottom left scenario (*u*_a_ = 0.01, *u*_w_ = 2 × 10^–5^), which has a total operation time of approximately 1 h. This illustrates that some periodic processes can take up to 10 min for the development of plug flow.

**Figure 7:**
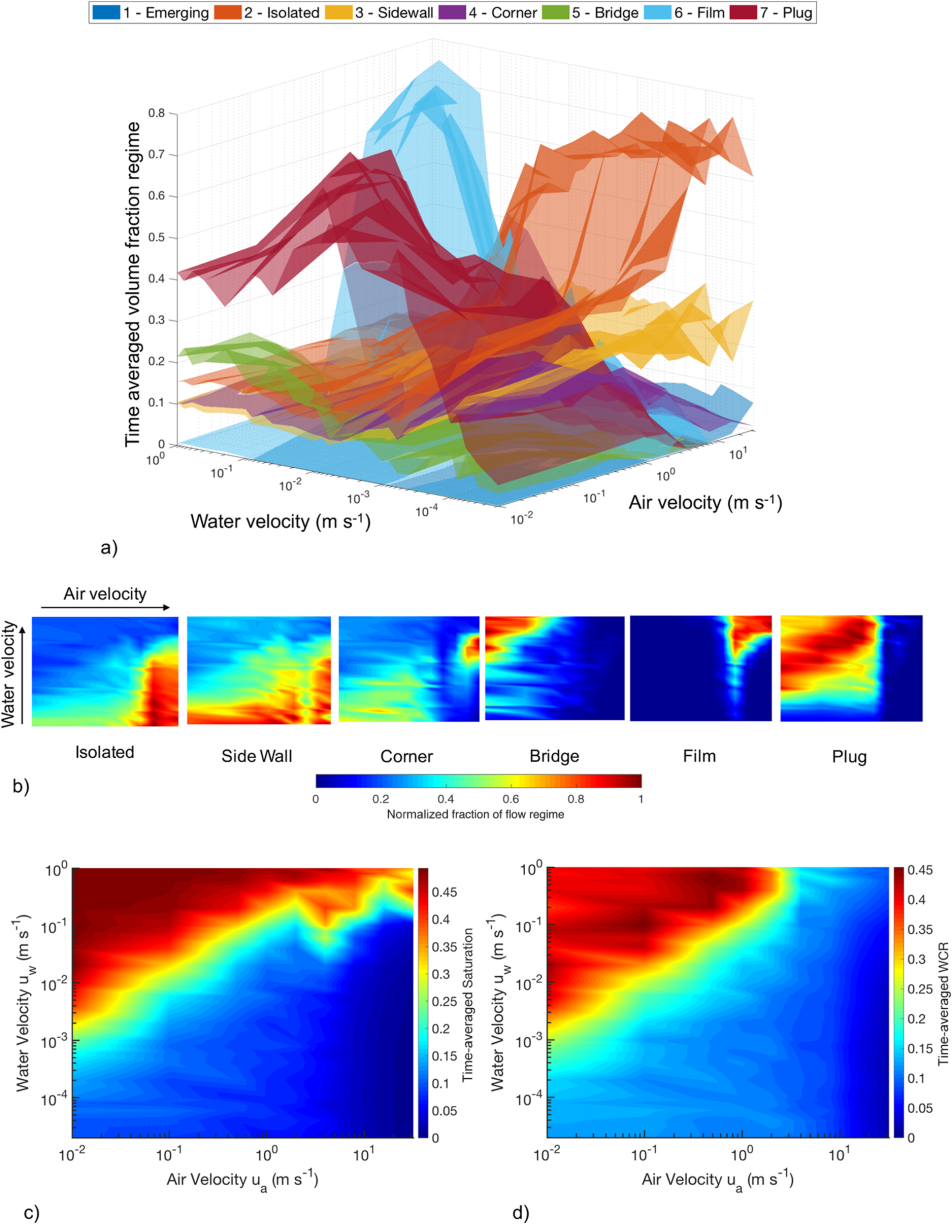
(a) Three-dimensional two-phase flow regime map for a fuel cell channel considering seven water regimes. Regime surfaces are triangulated based on time-averaged regime volume fraction ϵ across the 225 simulations shown in Supporting Information, Figure S1. (b) Saturation map with interpolated values between points, (c) WCR map with interpolated values between points. (d) Normalized fraction of the time-averaged water flow that is within each droplet regime.

Under typical PEFC operating conditions, the transition between two-phase flow regimes is caused by the difference in air velocity rather than the increase in the water flow rate through the same number of water clusters in the GDL. This conclusion was also reported in other regime maps proposed for fuel cell channels.^12,13^ Therefore, accumulation of water in the channel must be caused by increased discrete water sources such as condensation at higher current density. To further analyze each simulation case, the time-averaged regime, saturation, and WCR values were extracted to generate a three-dimensional two-phase flow regime map and the contour maps in Figure 7. The translation between water and air velocity to equivalent current density and stoichiometry is presented in Supporting Information, Figure S2a.

A three-dimensional two-phase flow regime map is required, resulting from the distribution of droplet configurations. Figure 7a shows that a distribution of flow regimes exists at each operating point. However, droplet regimes (emerging and side wall) consist of the majority of the flow at high air and low water velocity because detachment events occur with smaller droplet dimensions. This avoids the attachment to the top wall, which would lead to other flow regimes such as corner, bridge, and film flow.

Figure 7a suggests that characterization of two-phase flow regimes under specific conditions cannot be made subjectively.^5^ This result is highlighted by the transition region between plug flow and droplet flow, which contains different proportions of flow regimes. This transition region has a similar prediction window as results in experiments.^15^

The 3D regime map is decomposed into six regime maps in Figure 7b with the same water and air velocity axes instead of plotting the normalized fraction of each regime at each simulation point. This produces an intensity map, showing the regions of the operating space where each regime is more prevalent. The emerging regime is removed from this analysis because its presence is negligible and is only present for a very small amount of the simulation time.

The transition to film flow appears in the window of 1–3 m s^–1^ as shown in Figure 7b, which is similarly found in experimental results of ref (21). This also removes the water from the GDL channel surface, reducing the WCR shown in Figure 7d, where the highest fraction of film flow has a low WCR. This result suggests that as a minimum, the air velocity should be above this value to minimize water accumulation on the GDL surface.

The interaction between air and water velocity is more significant as water velocity increases (Figure 7c,d), where the channel becomes more saturated with a larger proportion of droplets covering the GDL surface. This also corresponds to an increased proportion of flow, that is, plug flow as shown in Figure 7b. The appearance of film flow occurs at a critical velocity, dependent on the channel properties, as expressed through the analytical equation developed in eq 42.

The DPM does not consider phase change and temperature effects. Therefore, the single-phase region shown in experiments^15,21^ is represented by droplet regimes. If phase change was included, there will be a distribution of evaporation rates along the channel, as shown by experiments and modeling,^56^ which should be considered in the next iteration of the model. Condensation effects may cause the distribution of injection points to move toward the side walls because water clusters under the rib regions will wick on the channel side walls, which was shown in X-ray imaging studies.^55^ Regardless, even without these physics introduced, the DPM can generally still predict the transition regions between plug, film, and droplet flows, as shown by the results of ref (15) visualized against the plug flow regime map in Figure S2b.

### Operating Fuel-Cell Regime Map

**Figure 8:**
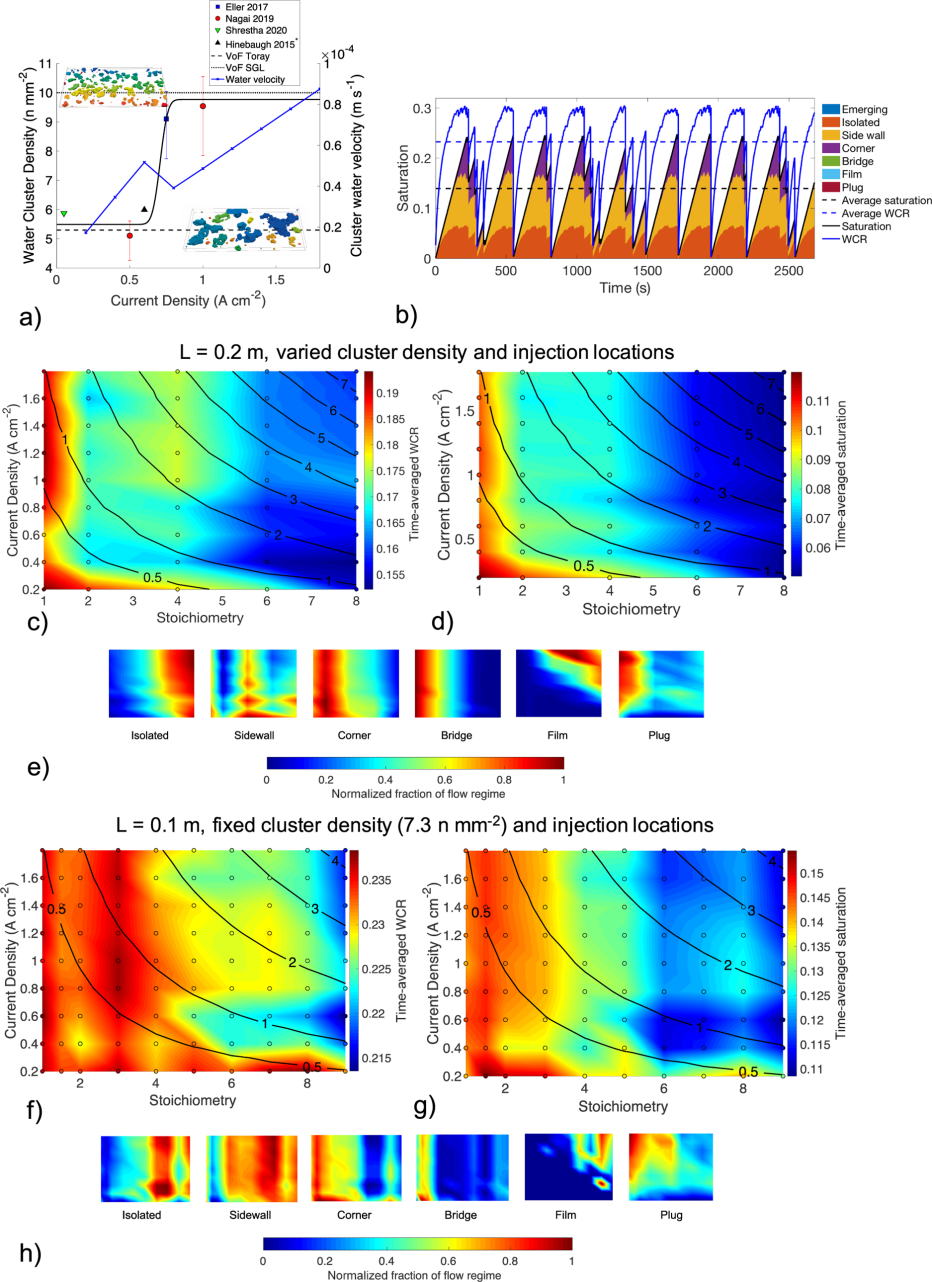
(a) water cluster density in the GDL extracted from the literature and VoF simulation with an error function fitted to the experimental points. Corresponding water velocity for L = 0.2 m study shown in blue. (b) Results of one simulation point (*i*_c_ = 1.4 A cm^–1^, ν = 4, and *L* = 0.1 m) highlighting the time-averaged method for the saturation and WCR maps. Time-averaged maps for scenario 1: (c) saturation, (d) WCR, and (e) normalized fraction of flow regime. Time-averaged maps for scenario 2: (f) saturation, (g) WCR, and (h) normalized fraction of the flow regime. Air velocity (m s^–1^) contours shown by black lines.

The water and air velocities shown in Figure 7 in PEFC are controlled by the stoichiometry and current density. The water cluster density in PEFC GDL was estimated from the published images in μCT studies. The water cluster density (n mm^–2^) was estimated at different current densities available from three different sources.^44,51,52,55^ Two additional VoF simulations were performed on μCT images of Toray (Toray TGP-H 060)^27^ and SGL (SGL 25 BA)^26^ materials for a full-area water injection to estimate the average cluster density, with cluster visualizations shown in Figure 8a. The VoF simulations represented an ex situ scenario, and therefore, the results do not represent a current density value. An error function was fitted to the extractions from the literature (*R*^2^ = 0.92) as
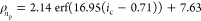
49

Equation 49 relates the water cluster density ρ_*n*_p__ (n mm^–2^) to the current density *i*_c_ (A cm^–2^). The average water cluster density across all data sets was 7.3 n mm^–2^. To investigate the cluster density correlation with current density, Figure 8c–e uses eq 49.

A series of 45 simulations was performed in the range of current density from 0.2 to 1.8 A cm^–2^ and stoichiometry from 1 to 8. Under the channel conditions used (L = 0.2 m, *H*, *W* = 0.001 m), this corresponded to a water and air velocity range of *u*_w_ = 2–9 × 10^–5^ m s^–1^ and *u*_a_ = 0.1–7.6 m s^–1^, respectively. The channel conditions used for scenario 1 (Figure 8c–e) were 1 mm × 1 mm × 0.2 m, θ_w_ = 70°, and θ_w_ = 120°. For scenario 2 (Figure 8f–h), the same conditions were used, instead with a fixed cluster density, spatial location, and shorter channel length (0.1 m).

**Figure 9:**
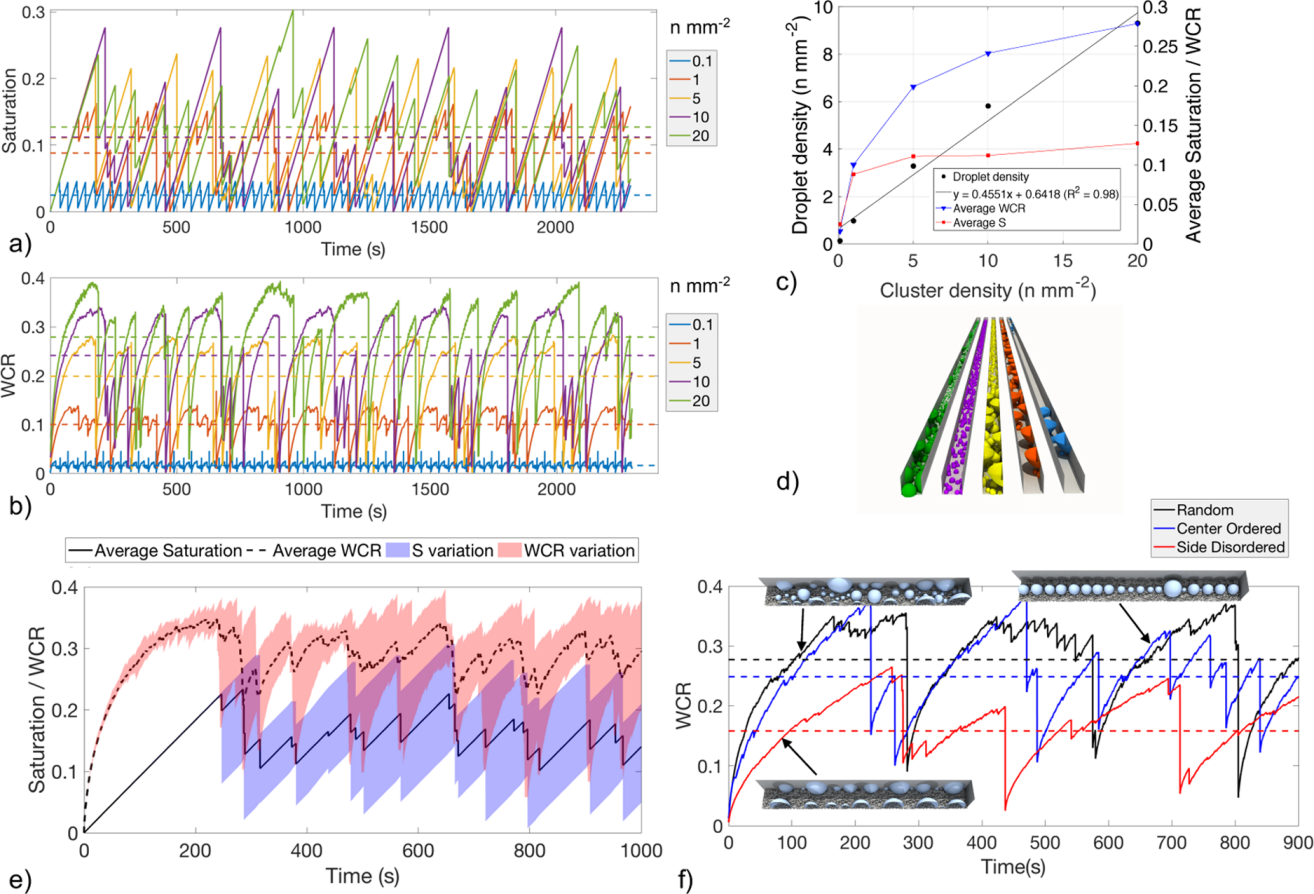
(a,b) Effect of GDL water cluster density (0.1–20 n mm^–2^) on channel saturation and WCR at 1.4 A cm^–2^ for the channel conditions in Figure 8. Dashed lines are the time-averaged values. (c) Relationship between average droplet density, average saturation, and WCR against cluster density. (d) Visualization of the last time step of each channel, with colors corresponding to the values in (a). (e) Deviation of saturation and WCR for five scenarios of randomly distributed injection locations. (f) WCR for random, ordered, and side-placed injection points.

Due to different realizations of water injection locations and the time averaging procedure shown in Figure 8b, there can be local fluctuations in the trend of the regime maps because the performance metrics are within similar magnitudes. An example of this is a channel scenario which has a distribution of GDL clusters situated close to the channel wall, which will exhibit a lower WCR, as is shown in Figure 9f. The local variations which do not agree with the trend of WCR, saturation, or regime maps are not representative, instead specific to the used water injection pattern and conditions.

To support this, for scenario 2, a set of 90 simulations with a shorter channel length (L = 0.1 m) was computed with a fixed cluster density of 7.3 n mm^–2^ and fixed spatial distribution of injection points shown in Figure 8f–h. This set of simulations was continued for 3 channel volumes of water injected, instead of 1 channel volume of water for the results in Figure 8c–e.

The water velocity *u*_w_ was determined using eq 43 and air velocity using eq 45. Figure 8c–e shows that the stoichiometry (air velocity) controls the channel saturation within the conditions tested, ranging from 0.05 at high air velocity and 0.12 at lower air velocity. However, critical two-phase flow regimes develop and appear for a short period of time compared to the overall simulation time, causing significant loss in saturation due to refreshing of the system of all droplets downstream. This can be visualized in Figure 8b by the peaks of saturation of 0.23 reducing sharply to 0.02 within a second of time.

Figure 8e shows the normalized regime maps for each regime. Plug flow was present in all cases, even at higher stoichiometry as a consequence of multiple coalescence events occurring along the channel length (0.2 m). However, its presence is higher at lower stoichiometry because the air velocity is lower, allowing greater volumes of water to appear prior to detachment. At high current density and stoichiometry, film flow developed resulting from larger external forces that cause deformation.^12^

The variation in averaged performance metrics could arise from the time averaging of dynamic data shown in Figure 8 or by variation of the cluster density and location. Scenario 2 shown by Figure 8f–h fixes the cluster density and location and simulates the process for 3 times longer results than the results in Figure 8c–e. The results show that the predicted regime map has a higher WCR and saturation which is related to the amount of droplet plugs refreshed in the system. A Longer channel length has a greater potential to refresh the system of more droplets, thus lowering these time-averaged properties.

The prevalence of performance critical regimes (plug, film, and bridge) are generally the same between the two scenarios of Figure 8e,h. However, the differences are related to the change in the channel length and possibly the extended simulation time. The air velocity range is different in scenarios 1 and 2 (due to the change of active area), meaning that there will be a change in time-averaged metrics. Although local variations are still shown in Figure 8f–h, the variation between surrounding points is small (<0.01 WCR or saturation). This difference this can be attributed to the difficulty in representing dynamic data as a single time-averaged result.

The operating current density regime maps shown in Figure 8 results should be considered with caution since first, the water cluster density correlation in Figure 8a may not be correct and requires further study. Second, many physical phenomena such as phase change have not been implemented in the current version of the model. During in situ operation evaporation of droplets, the inlet of the channel will experience a lower or even suppressed flow rate.^56^

The run time for the DPM is highly dependent on the number of injection locations and droplets in the channel. As the channel length increases, each droplet must track each injection pore to maintain mass conservation, especially during plug flow. In total, the 45 simulation cases in Figure 8c–e required 10 h of operation time (1 channel volume of water injected), which required 25 h of CPU time. Future work will improve this algorithm for faster run times.

### Effect of Water Cluster Density and Distribution

Under the same operating conditions, the water cluster spatial density and distribution can affect the evolving flow regimes, WCR, or saturation. To test the robustness of the model and to further understand the local minima exhibited in Figure 8, different simulation scenarios were set up, with results shown in Figure 9.

Figure 9a–d uses a 0.1 m channel with varied water cluster density (0.1–20 n mm^–2^, *i*_c_ = 1 A cm^–2^ and ν = 2) for a longer simulation period of 3 channel volumes injected. The results show that increasing the water cluster density increases both the saturation and WCR significantly, suggesting that to reduce water accumulation in the channel, the number of discrete water sources in the GDL should be minimized. This could be possible by optimization of microporous layer defects^26^ or through the already present condensation processes, which increases the number of water clusters emerging from the rib–GDL interface.^57^

Figure 9c averages the number of droplets, saturation, and WCR over the simulation time for the corresponding cluster density. Increasing the water cluster density increases each of these metrics and there is an approximate relationship between the number droplets in the channel and number of clusters in the GDL. This linear relationship was used to estimate the cluster density in the GDL, assuming that data for droplet density in the channels are known. This relationship was used on the data from ref (55) shown in Figure 8a.

Average saturation plateaus with cluster density. Dense regions of injection points will form many smaller droplets which coalesce to one larger droplet at equivalent water velocity of the sum of all injection points. This essentially acts as a single source, thus limiting the growth of droplets from additional points.

Figure 9d shows the distribution of droplets for the last time step of each of the cluster density simulations in Figure 9a,b, with the color corresponding to the cluster density used. Depending on where the critical plug, film, or bridge flow occurs, generally droplets downstream from that location will be flushed of droplets. Since phase-change processes are not introduced, this means that the start of the channel is likely to be more saturated than downstream.

Furthermore, the spatial distribution of injection locations (i.e., closeness of injection locations to each other and to the walls) can alter the channel performance metrics. This analysis is shown in Figure 9e where five simulations at the conditions L = 0.01 m and *i*_c_ = 1 A cm^–1^ with 139 injection points were repeated but with different randomized spatial locations. The variation between each simulation for saturation (blue) and WCR (red) from the averaged values is significant with a variation of 0.12–0.13 saturation and WCR from the averaged value. A scenario with injection locations distributed on average closer to the channel walls will experience a lower average WCR, as shown in Figure 9f. This can be visualized by the 3D rendering of the last time step of the three distribution scenarios.

This analysis suggests that water cluster emergence close to the channel walls will facilitate water removal toward the walls, allowing less area restriction for diffusion into the GDL from the channel. The results illustrating the effect of geometry variation from Figure 9 also provide a possible reason for the local minima in the WCR shown in Figure 8c–e. In new material design, it is important to consider the effect of controlling the injection locations (such as through laser perforation^58^ or 3D printed designs^59^ that will produce ordered injection points) on the channel performance metrics.

## Conclusions

A DPM was developed to predict and elucidate the effect of operating conditions on two-phase flow regimes in gas channels in fuel cells. The modeling principles were validated against VoF simulations. Given the simplicity, it can efficiently simulate two-phase flow in a channel to generate two-phase flow regime maps. The evolution of water in the channel system exhibits dynamical chaos resulting from the interconnected temporal and spatial events. The following two-phase flow regimes were simulated in the DPM: emerging, isolated, side wall, corner, capillary bridge, film, and plug flows.

Through a series of mechanistic approximations, the model could estimate droplet coalescence and wall attachment mechanisms occurring in this study:

1. Water–air two-phase flow with coalescence and wall attachment can be predicted using the DPM, validated against VoF simulations. This had an acceptable predictive ability, with an approximate ×200,000 increase in simulation speed.
2. Channel wall wettability and number density of water clusters in the GDL can alter the two-phase flow regime, channel saturation, and WCR. Hydrophilic walls promote growth and spreading of water into the corners, reducing the WCR. Hydrophobic walls produce droplets covering the GDL surface and the channel cross section more significantly, leading to greater pressure loss.
3. The increase in saturation of the channel at a higher current density is suggested to be related to an increase in the water cluster density emerging from the GDL and not by the increase in the water flow rate through pathways already present.
4. Development of liquid slugs, plugs, and films only occurs in certain airflow rates as a consequence of multiple droplet coalescence along the channel. These regimes develop large pressure drop fluctuations in the air, leading to greater power required by the compressor. Due to their size relative to the size of the channel, the dynamics of these regimes removes the majority of droplets downstream, removing a significant amount of water from the channel (at the expense of large pressure drop), causing periodic reduction in saturation.
5. The water emergence, accumulation, and removal process from the channel is periodic; channel performance metrics fluctuate around a steady value. However, at low water flow rates such as those experienced during fuel cell operation, the time between significant removal cycles can be several minutes to hours. During this time, a developed plug may only stay in the system for several seconds depending on the air velocity and length of the channel.

Accelerated water flow rates used in computational studies cannot be used to replicate the two-phase flow regimes in the channel during PEFC operation because saturation, the WCR, and regime fractions are different. Under PEFC conditions, coalescence events can cause plug flow formation, resulting in variation in the WCR, which will in turn affect oxygen transport. It is possible that results shown in this study are only representative two-phase flow in a single PEFC channel up to 0.2 m in length without phase change and flow through the GDL.

Small perturbation in initial conditions such as water cluster emergence locations can cause differences in process dynamics. This, along with the errors involved with time averaging of the cyclical data, can cause local deviations in the saturation, water coverage, and regime maps. Therefore, each regime map is specific for each case studied, changing the wettability or length of the channels studied, and will change the maps produced.

Future iterations of the DPM should include species transport in the channel and porous regions, as well as including different flow field designs. Extension of the water regimes should include droplet attachment to the top wall, which will use the same equations as the isolated regime. Temperature effects including phase change can be accounted for using an energy balance around each droplet. Considering that the DPM has access to interface properties, mass transfer in a network of hydrodynamic channels which solves the velocity distribution of air will be able to achieve this goal. Active fuel cell operation can be introduced by including a volume-averaged approach for flow and transport in porous media coupled to the channel network similar to that of ref (49). Phase change effects and flow field consideration may alter the two-phase flow characteristics, which is the direction of our future work.

## Supporting Information Available

Full regime map used for sensitivity analysis of Figure 7, regime map translation for Figure 7 and comparison to ref (15), details for the procedure of force extraction from CFD, additional force prediction comparison between DPM and VoF, and additional details for droplet regime derivations (PDF)

## Nomenclature

*A*_c_: droplet cross-sectional area [m^2^]
*A*_s_: droplet interfacial area [m^2^]
*A*_s,l_: solid–liquid contact area [m^2^]
*A*_surf_: area of water covering the GDL surface [m^2^]
α_w_: angle between walls and the droplet center of volume [rad]
*b*: medial axis of air between the channel walls and droplet [m]
ϵ: volume fraction of droplets in the droplet regime classification
*f*: air velocity acceleration factor
*F*_D_: drag force [N]
*F*_σ_: adhesion force [N]
*F*_I_: inertial force [N]
*F*_μ_: viscous shear force [N]
*F*_μ,s_: viscous shear force between water and walls [N]
Faraday constant [C mol^–1^]
*h*: height of the droplet [m]
*H*: channel height [m]
*I*: current [A]
*i*_c_: current density [A cm^–1^]
*L*: channel length [m]
*L*_*i*_: droplet length along the channel [m]
*L*_σ_: liquid–solid contact line length normal to airflow [m]
*m*: droplet mass [kg]
*M*_w_: molecular weight of water [kg mol^–1^]
μ: fluid dynamic viscosity [kg m^–3^]
*n*: number of droplets, injection points, or regimes
ν: stoichiometry
*p*: pressure [Pa]
*P*_in_: inlet pressure [Pa]
ρ: fluid density [kg m^–3^]
ϕ: ratio of the circular segment to circle area
*Q*_a_: volumetric flow rate of air [m^3^ s^–1^]
*Q*_w_: volumetric flow rate of water [m^3^ s^–1^]
*R*_c_: radius of curvature [m]
*R*_con_: contact radius [m]
*R*_g_: universal gas constant [J mol^–1^ K^–1^]
*S*: sink or source term
σ: air–water interfacial tension [N m^–1^]
*t*: time [s]
*T*: inlet temperature of air [K]
θ_R_, θ_A_, θ_app_: advancing, receding, and apparent contact angle [rad]
θ_s_, θ_w_: GDL and channel wall contact angles [rad]
*u*_a_: inlet velocity of air [m s^–1^]
*u*_a_^*^: average velocity of air in the section above droplet [m s^–1^]
*u*_w_: velocity of water [m s^–1^]
*V*_*i*_: volume of the droplet *i* [m^3^]
*W*: channel width [m]
*x*_O_2__: inlet mol fraction of oxygen in air
*x*, *y*, *z*: Cartesian coordinates

## Subscripts

a: air
w: water
*i*: droplet *i*
*i*: droplet *j*
p: water injection point
p, *i*: pore p to droplet *i* connection
*i*, *r*: droplets *i* in regime *r*
*ij*: connection between droplet *i* and *j*
w_*i*_: water to droplet *i*
