# Supplementary material for: Discrete-Particle Model to Optimize Operational Conditions of Proton-Exchange Membrane Fuel-Cell Gas Channels
Source: ACS Appl Energy Mater. 2021 Sep 22;4(10):10514–33. doi: 10.1021/acsaem.1c01391 (PMC8552217; doi:10.1021/acsaem.1c01391)
Supplement: Supplementary file 1 — ae1c01391_si_001.pdf [file ae1c01391_si_001.pdf]

Supporting Information

A discrete-particle model to optimise operational conditions of PEM fuel cell gas channels

Daniel Niblett, Stuart Martin Holmes, Vahid Niasar*

Department of Chemical Engineering and Analytical Science, University of Manchester, Manchester, M13 9LP

*corresponding author: vahid.niasar@manchester.ac.uk

Full regime map sensitivity analysis

The highlighted results presented in Figure 6 are taken from the set of 225 simulation cases as shown in Figure S1.

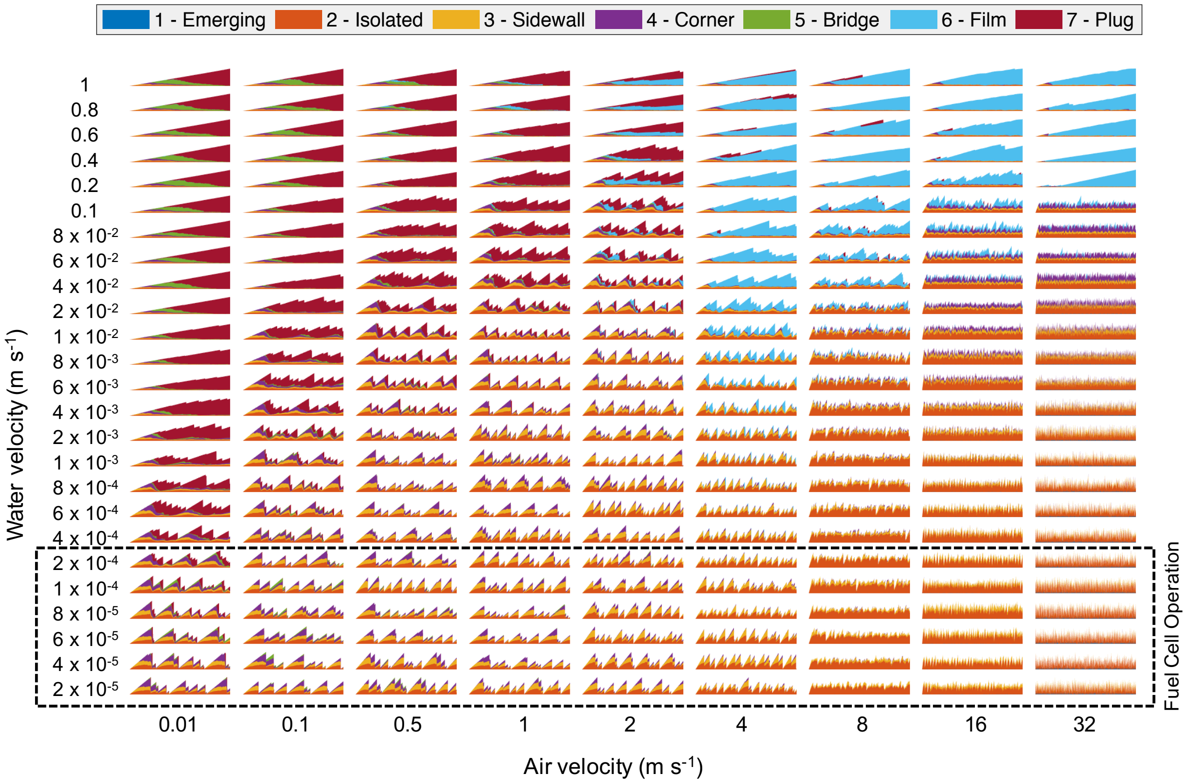

Figure S1: Saturation and regime profiles for 225 simulation cases, varying air velocity and water velocity with values corresponding to marker (x,y) location. Channel dimensions (1 mm × 1 mm × 0.2 m) for the simulation period of one channel volume injection with water injection from n_p=400. Fuel cell operating space marked out on the regime map by dashed box corresponding to between 0 - 1.2 A cm^{-2}.

Regime map translations

The presented Figure S2 (a) in this section contain the translation between water and air velocity to equivalent current density and Stoichiometry for the channel conditions specified for Figure 7. The region of current density and

Stoichiometry investigated in Figure 8 is also highlighed in red, although water velocity will be different due to different water cluster density used. Figure S2 (b) contains the transition lines from the experimental results of Ref[15] on the normalised fraction of plug flow regime map taken from Figure 7 (b).

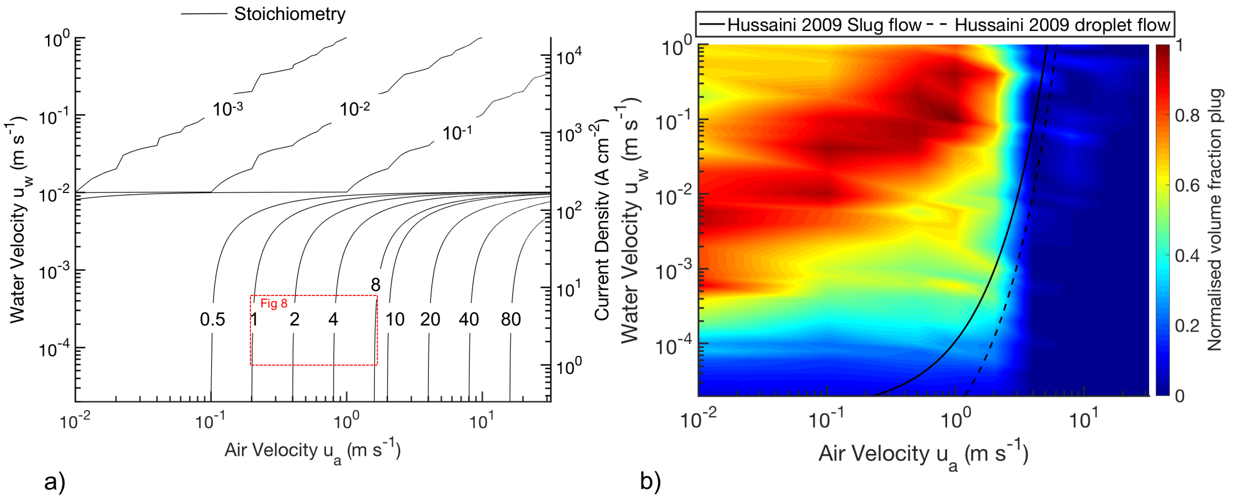

Figure S2: a) operating condition translation factor for the regime maps shown in Figure 7. Current density scales linearly with water velocity at constant water injection conditions. Iso-lines of equivalent Stoichiometry are presented for reader conversion. The red region highlights the possible range investigated in Figure 8 but with different channel conditions. b) normalised fraction of plug flow map with the transition lines of plug flow and droplet flow from Ref[15] applied.

Extraction of forces from CFD

Droplet detachment in a channel was simulated using the volume of fluid (VoF) method on a three-dimensional domain (625,000 cells, $\Delta x = 20$ μm) shown in Figure 2 (b). Forces acting on the droplet over time was extracted. This information was used as semi-validation, where experimental data for forces acting on discrete droplets is difficult to obtain. In VoF, both phases share the

same pressure field, separated by a interface with a specific volume thickness. To remove the effect of the capillary pressure on the air pressure drop in the channel, the interface was constructed based on a contour 0.01 alpha (volume fraction water). The forces were extracted using Paraview using the following methods[60].

The inertial force $F_{p,x}$ was found by integrating the pressure on the interface over the area normal to the direction of flow (x direction):

$$F_{p,x} = \int_{A_x} n_x p_x dA_x \tag{S1}$$

The viscous shear force $F_{\mu,x}$ was found by calculating the gradient of velocity in the entire domain, where at the interface between water and air the gradient of velocity was multiplied by the viscosity of air to find the shear stress acting on the surface in the two directions (τ_{xz}, τ_{xy}) normal to the direction of flow:

$$\tau_{xz} + \tau_{xy} = \mu \left(\frac{du_x}{dz} + \frac{du_x}{dy} \right) \tag{S2}$$

The shear stress was integrated over the interface surface area normal to the gradient of velocity in the component direction to find the shear force:

$$F_{\mu,x} = \int_A \mu n_y \cdot \nabla u_x dA + \int_A \mu n_z \cdot \nabla u_x dA \tag{S3}$$

The adhesion force $F_{\sigma,x}$ was found by extracting the solid-liquid-air contact line and integrating the normals with respect to the direction of flow, multiplied by

the contact angle θ with respect to the z direction (channel height direction).

$$F_{\sigma,x} = \int_{L_\sigma} \sigma n_x \cos\theta dL_\sigma \tag{S4}$$

Force comparison between DPM and VoF for capillary bridge

For three different volume water droplets in the capillary bridge regime, Figure S3 shows the comparison of forces and interface shapes predicted DPM and VoF models. The shear force, inertial force, adhesion force and air pressure drop was extracted using the same method as above at one time step for each case. The magnitude of the forces predicted by each method are of similar values for each force. The three-dimensional representation used for the coalescence algorithm matches the dimensions of capillary bridges produced by the interface resolving VoF method at different wall contact angles (except form the spherical coordinates that is used for the capillary bridge in the DPM model). This analysis, combined with the comparison against transient VoF data in Figure 4 provided confidence in the predictions of momentum exchange between air and water droplets of different geometry.

Derivation of Force Balance

The equations used to estimate the forces presented in Section which act on different droplet geometry were approximated from first principles using the following approaches.

Inertial Force

The obstruction of air flow by droplets in the channel causes acceleration of flow, converting pressure energy into kinetic energy resulting in pressure loss. Using the conservative form of the Bernoulli equation, the pressure drop was estimated between point 1 (before the droplet) and point 2 (at the droplet cross section)[61]:

$$\frac{p_1}{\rho} + \frac{u_1^2}{2} + gz_1 = \frac{p_2}{\rho} + \frac{u_2^2}{2} + gz_2 \tag{S5}$$

where p is the pressure, u is the velocity, z is the height coordinate and g is the gravitational acceleration. Equation (S5) is reformulated, ignoring gravity to obtain the pressure drop between points 1 and 2:

$$\Delta p_i = \frac{\rho}{2}\left(u_2^2 - u_1^2\right) \tag{S6}$$

The velocity directly above the droplet u_2 was determined using the continuity equation for air flow at a constant flow rate where u_1 is equal to the average velocity of air supplied at the inlet to the channel:

$$u_2 = \frac{u_a HW}{HW - A_c} \tag{S7}$$

The inertial pressure in eq. (S6) acts on the interface area of the droplet normal to the direction of flow A_c:

$$F_I = \Delta p_i A_c \tag{S8}$$

Viscous Shear Force

The shear stress τ_{zy} acting on the acting on the air-water interface area A_s is found by the viscosity of air multiplied by the gradient in velocity in the y-direction, evaluated at the droplet surface ($y = 0$):

$$\tau_{zy} = \mu \frac{du_z}{dy} \tag{S9}$$

The flow of air in the channel section above the droplet is estimated as planar Poiseuille flow, with the average velocity $\bar{u}$:

$$\bar{u} = \frac{H^2}{12\mu} \left(-\frac{dp}{dz} \right) \tag{S10}$$

where H is the height of the channel section between the droplet and the top channel surface. The shear stress on the droplet surface is therefore estimated as:

$$\tau_{zy} = \frac{H}{2} \left(-\frac{dp}{dz} \right) \tag{S11}$$

The fluid-fluid interfacial area A_s determined through analytical approximation is used to calculate the shear force where the average velocity above the droplet is $\bar{u} = u_2$:

$$F_\mu = \frac{3\mu u_2}{b} A_s \tag{S12}$$

where b is the approximate thickness of the viscous boundary layer in laminar flow, estimated based based on the droplet configurations.

Derivation of Droplet Regimes

In this section, additional explanation and equations are presented for the regimes schematics presented in Figure 3.

Regime 1 - Emerging

Equation (13) is solved using an Newton iterative method to find the height of the droplet based on a known contact radius R_p:

$$h^3 + 3R_p^2 h - \frac{6V_i}{\pi} = 0 \tag{S13}$$

The air medial axis position b shown in Figure 3 (a) is:

$$b = \frac{H - h}{2} \tag{S14}$$

The area of water that covers the GDL surface, used to determine the water coverage ratio and the liquid-solid shear force was calculated using the radius of the pore:

$$A_{surf} = \pi R_p^2 \tag{S15}$$

Regime 2 - Isolated

In contrast to regime 1, the contact radius on the GDL surface increases with droplet volume and was calculated using the radius of curvature from eq. (19):

$$R_{con} = R_c \sin(\pi - \theta_s) \tag{S16}$$

The height of the droplet was found using:

$$h = R_c(1 - \cos(\theta_s)) \tag{S17}$$

The area of water covering the GDL A_{surf} and air medial axis location b are calculated using eq. (S15) and (S14) respectively.

Regime 3 - Side Wall

Using trigonometric identities and equations for volume of spherical segments, the dimensions in Figure 3 (c) was estimated. The length from the geometric center to the channel wall x_1 is:

$$x_1 = R_c \sin(\alpha_w) \tag{S18}$$

The distance from the channel wall to the droplet interface x_2 is:

$$x_2 = x_1 - R_c \tag{S19}$$

The distance of the geometric center to the GDL surface x_3 is:

$$x_3 = R_c \sin(\alpha_2) \tag{S20}$$

The distance from the geometric center to the top of the GDL interface on the side wall of the channel x_4 is:

$$x_4 = \sqrt{R_c^2 - x_1^2} \tag{S21}$$

The distance of the interface on the GDL surface to the channel wall x_5 is:

$$x_5 = \frac{x_3}{\tan(\theta_s - \frac{\pi}{2})} - x_1 \tag{S22}$$

The angle between the geometric center and the top interface point on the channel side wall α_w is:

$$\alpha_w = \frac{\pi}{2} - \theta_w \tag{S23}$$

The angle at the geometric center, between the points at the top of the side wall and on the GDL surface θ_c is:

$$\theta_c = 2(\pi - \theta_s) \tag{S24}$$

The volume of this shape is determined by truncating the volume of a spherical cap:

$$V = \frac{\pi R_c^2}{3}(3R_c - h) \tag{S25}$$

where the height of the droplet h in this scenario is x_2 shown in Figure 3 (c) and is related to the radius of curvature using:

$$x_2 = R_c(1 - \cos(\alpha_w)) \tag{S26}$$

The spherical cap is truncated by the GDL surface and can be approximated by the ratio of areas between a circular segment with an internal angle of θ_c and a circle with radius R_c, this ratio ϕ_1 is:

$$\phi_1 = \frac{\theta_c - \sin\theta_c}{2\pi} \tag{S27}$$

Combining eq. (S25) and (S26) produces the droplet volume, expressed as a function of R_c:

$$V_i = \frac{\pi R_c^3}{3}(2 - \sin(\alpha_w) + \sin^3(\alpha_w)) \tag{S28}$$

The radius of curvature is found by multiplying eq. (S28) by the area ratio ϕ_1 in eq. (S27):

$$R_c = \left(\frac{3V_d}{\pi(2 - 3\sin(\alpha_w) + \sin^3(\alpha_w))\left(1 - \frac{\theta_c - \sin(\theta_c)}{2\pi}\right)} \right)^{\frac{1}{3}} \tag{S29}$$

The cross section area of the droplet was determined using equations for the circular and triangular cross-sections shown in Figure 3 (c):

$$A_c = \frac{R_c^2(\theta_c - \sin(\theta_c)) + x_5(x_3 + x_4)}{2} \tag{S30}$$

The surface area of the droplet was determined as the spherical cap surface area multiplied by the area ratio ϕ_1. The area of the GDL covered by water is determined using the dimension x_5 as the height of a circular segment with central angle of $2\theta_w$. The radius corresponding to this circular segment is found

by:

$$R_{con} = \frac{x_5}{1 - \cos(\theta_w)} \tag{S31}$$

Therefore, the area of the GDL that the droplet covers was determined as:

$$A_{surf} = \frac{R_{con}^2(2\theta_w - \sin(2\theta_w))}{2} \tag{S32}$$

The air medial axis was approximated by the difference between x_2 and the width of the channel W.

$$b = \frac{(W - x_2)}{2} \tag{S33}$$

Regime 4 - Corner Droplet

The corner drop is approximated as a spherical cap with contact angle θ_w, truncated by the channel corner. The dimensions in Figure 3 (d) are as follows. The angle from the interface at the channel wall to the interface geometric center is:

$$\alpha_w = \frac{\pi}{2} - \theta_w \tag{S34}$$

The distance from the geometric center to the channel wall is:

$$x_1 = R_c \sin(\alpha_w) \tag{S35}$$

The distance from the channel wall to the interface maximum across the channel width is:

$$x_2 = R_c - x_1 \tag{S36}$$

The volume of the corner droplet was determined by combining eq. (S27) and (S25):

$$V = (2R^3 - 3R^2x_1 + x_1^3)\frac{(2\theta_w - \sin(2\theta_w)}{6} \tag{S37}$$

The radius of curvature R_c was determined as a function of droplet volume and the contact angle of the wall shown. The air medial axis is found using eq. (S33).

Regime 5 - Truncated Capillary Bridge

The dimensions in Figure 3 (e) were used to derive equations for the two radius of curvature. The volume of this shape was found by the addition of two volumes, firstly the volume of a cylindrical shell between R_2 and R_3, multiplied by the area fraction occupied by water at the cross section Wz_1. Secondly, the volume of a cylinder multiplied by the area ratio at the channel walls. The radius of curvature of the internal curvature R_1 is:

$$R_1 = \frac{W}{2\cos(\theta_w)} \tag{S38}$$

The distance between R_3 and R_2 is:

$$z_1 = R_1\left(1 - \cos\left(\frac{\pi}{2} - \theta_w\right)\right) \tag{S39}$$

Due to the curvature of the interface, the area comprised of liquid within Wz_1 is estimated using the area ratio ϕ_1, where $\theta_1 = \pi - 2\theta_w$:

$$\phi_1 = \frac{R_1^2(\theta_1 - \sin\theta_1)}{2Wz_1} \tag{S40}$$

The next clipping stage involved the truncation of the volume of the cylinder V_{R_2} by the channel location, which is determined by the wall contact angles. The secondary area ratio ϕ_2 is defined with a central segment angle of circle 2, where $\theta_2 = 2\theta_w$.

$$\phi_2 = \frac{\theta_2 - \sin\theta_2}{2\pi} \tag{S41}$$

The equation for the volume of a truncated capillary bridge was determined a function of the three cylindrical volumes:

$$V_i = \phi_2(V_{R_3} + \phi_1(V_{R_2} - V_{R_3})) \tag{S42}$$

Following each cylinder has a volume $V = \pi R^2 W$, eq. (S42) is:

$$V_i = \phi_2(\pi R_3^2 W + \phi_1(\pi R_2^2 W - \pi R_3^2 W)) \tag{S43}$$

Using the schematic in Figure 3 (e) where $R_3 = (R_2 - z_1)$, the full equation becomes:

$$V_i = \phi_2 \pi W R_2^2 - 2\phi_2\phi_1\pi z_1 W R_2 + \phi_2\phi_1\pi z_1^2 W \tag{S44}$$

This quadratic equation was solved using the quadratic formula to determine R_2:

$$R_2 = \frac{-2\phi_2\phi_1\pi z_1 W + \sqrt{(-2\phi_2\phi_1\pi z_1 W)^2 - 4(\phi_2\pi W)(\phi_2\phi_1\pi z_1^2 W - V)}}{2(\phi_2\pi W)} \tag{S45}$$

The height of the droplet in the direction of the channel height h at the side walls (R_2) and in the middle of the channel (R_3) is determined:

$$h = R_c(1 - cos(\theta_w)) \tag{S46}$$

Regime 6 - Film

This shape is comprised of a rectangular section shown in Figure 3 (f), with length L_i spanning the width of the channel W and two truncated cylindrical sements. The rectangular volume, has an internal curvature determined by the distance between the two side walls as in Figure 3 (e):

$$V_{R_1} = \left(hW - \frac{R_1}{2}(\theta_w - \sin\theta_w)\right) L_i \tag{S47}$$

The internal radius of curvature is found using eq. (S38). The volume of the two end segments V_{R_2} is found by the circular segment area formed by the contact angle and $\frac{\pi}{4}$(parallel to the channel walls):

$$V_2 = (hx_1 + R_2(\theta_2 - \sin\theta_2)) \tag{S48}$$

where x_1 is the distance on the channel wall to the normal of the radius and $\theta_2 = \left(2\theta_w - \frac{\pi}{2}\right)$. The radius R_2 is found by:

$$R_2 = \frac{h}{1 - \cos\left(\theta_w\right)} \tag{S49}$$

Combining eq. (S47) and (S48), the length of the film is estimated as:

$$L_i = \frac{V_i - (hx_1 + R_2(\theta_2 - \sin\theta_2))W}{(hW - \frac{R_1}{2}(\theta_1 - \sin\theta_1))} \tag{S50}$$

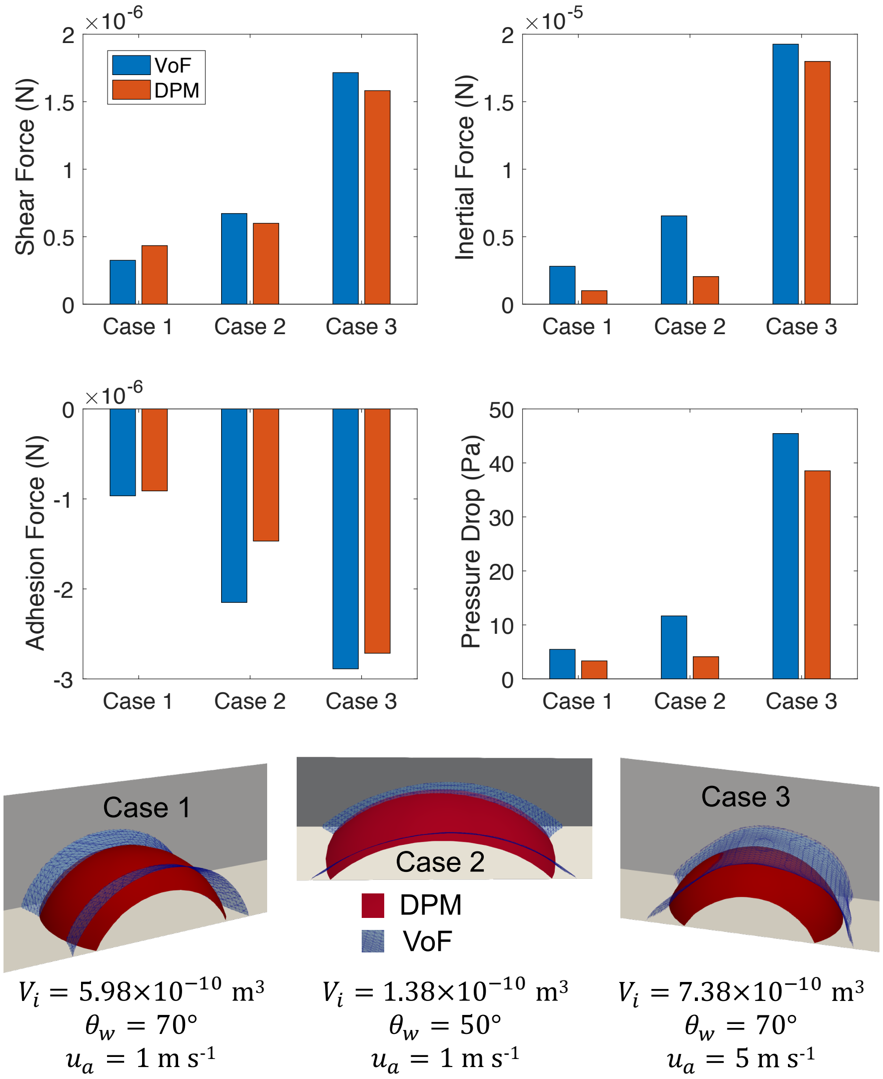

Figure S3: Comparison of shear force, inertial force, adhesion force and pressure drop for three different capillary bridge cases predicted by VoF and the DPM model. Model outputs for the interfaces of the VoF (blue) and DPM model (red) at different conditions provided.
